# Global burden and cross-national inequalities of tobacco-attributable cancers in adults aged 40 and above, 1990–2021: a population-based study

**DOI:** 10.3389/fonc.2025.1631356

**Published:** 2025-07-25

**Authors:** Yi Li, Hui Li, Lirui Tang, Chuanben Chen, Chaoxiong Huang

**Affiliations:** Department of Radiation Oncology, Clinical Oncology School of Fujian Medical University, Fujian Cancer Hospital, Fuzhou, China

**Keywords:** tobacco exposure, cancer burden, global burden of disease (GBD) study, crossnational inequalities, sociodemographic index (SDI), disability adjusted Life Years (DALYs), deaths

## Abstract

**Background:**

Tobacco exposure substantially increases the global cancer burden; however, studies targeting specific subgroups are scarce. We aimed to investigate global burden trends of tobacco-attributable cancers among people aged ≥40 and the associated cross-national inequalities based on the sociodemographic index (SDI).

**Methods:**

We performed secondary analyses on data from the Global Burden of Disease (GBD) Study 2021. The global tobacco-attributable cancer burden was assessed by age-standardized (ASR)-disability adjusted life years (DALYs) and deaths. The estimated annual percentage changes were used to illustrate temporal global and regional trends from 1990 to 2021. Decomposition analyses determined the impact of population growth, aging, and epidemiological changes on disease burden. The slope inequality index (SII) and concentration index (CI) were used to quantify cross-country inequalities in the tobacco-attributable cancer burden.

**Results:**

In 2021, global tobacco-attributable ASR-DALYs among people aged ≥40 were 1,687.49 per 100,000 people, a continuous decline since 1990, and ASR deaths were 72.36 per 100,000 people. By 2030, they are projected to fall to 1,464.68 and 64.59 per 100,000, respectively. Men exhibited higher DALYs and deaths than women (40.8 million DALYs, 1.7 million deaths). The most prominent tobacco exposure was smoking (ASR-DALY: 1,603.98/100000). Among the 16 cancers observed, tracheal, bronchial, and lung cancers had significantly higher ASR-DALYs and ASR-related deaths than other cancers. Population growth was the main cause of the tobacco-attributable cancer burden, followed by epidemiological changes. The highest ASR-DALYs and deaths were observed in the medium-high SDI regions and the lowest in the low SDI regions. Health inequality analyses showed that the DALYs SII declined from 2,654/100,000 in 1990 to 1,178/100,000 in 2021; however, the difference between high and low SDI countries narrowed significantly. The DALYs CI was 0.17 in 1990 and 2021, and the mortality CI increased from 0.17 to 0.18.

**Conclusions:**

The cancer burden attributable to tobacco use varied significantly according to sex, age, region, and SDI. The global tobacco-attributable cancer burden among people aged ≥40 has been declining since 1990, paralleling mitigated yet persistent cross-national inequalities. The study’s findings could help to develop strategies for improving the prevention and treatment of cancers.

## 1 Introduction

Cancer is one of the leading contributors to the disease burden and mortality worldwide. According to the Global Burden of Disease (GBD) 2022 study, cancer causes nearly 10 million deaths annually, accounting for 16.8% of all deaths worldwide (1). As the population ages and life expectancy increases, the incidence of cancer continues to rise, with a projected 77% increase in cancer cases globally by 2050 compared to 2022 (2). Cancer threatens individual health and puts heavy pressure on socioeconomic systems. For example, direct cancer-related healthcare costs and productivity losses amounted to US$1.16 trillion globally in 2019; this figure is growing exponentially in the context of an aging population (3). Therefore, cancer prevention is a global public health priority. According to the World Health Organization (WHO), approximately 30–50% of cancers can be effectively prevented by reducing risk factors and early screening (4), and tobacco control is considered one of the most cost-effective interventions.

Tobacco use is the most important modifiable risk factor for cancer and is strongly associated with at least 20 cancer types (5). It is the leading cause of lung cancer, accounting for more than 85% of lung cancer cases globally, and it significantly increases the risk of oral, laryngeal, esophageal, and bladder cancers (6, 7). In addition, the use of non-combustible tobacco (e.g., chewing tobacco) is strongly associated with oral and pancreatic cancers, particularly in South and Southeast Asia (6). Secondhand smoke exposure is also non-negligible and leads to approximately 120,000 lung cancer deaths annually, with women and children being the most at-risk groups (8). According to the GBD 2019 study, approximately 22% of cancer deaths globally are attributed to tobacco use, a proportion that has declined in high sociodemographic index (SDI) countries but continues to rise in low and medium SDI countries (9). The widespread prevalence of tobacco and the multi-target nature of its carcinogenic mechanisms have contributed to key breakthroughs in cancer prevention.

The burden of cancer caused by tobacco use is highly preventable. Studies have shown that the full implementation of the World Health Organization Framework Convention on Tobacco Control (WHO FCTC) policies (e.g., higher tobacco taxes, bans on advertising, and smoking in public places) could prevent more than 100 million tobacco-attributable deaths in 30 years (10). Although studies have been conducted to analyze the impact of tobacco use on the global cancer burden from 1990 to 2019 (11), studies targeting specific subgroups (e.g., adults aged 40 and above) remain insufficient. As the latency period for tobacco carcinogenesis is up to 20–30 years, the cancer burden in middle-aged and older populations might better reflect historical tobacco epidemic trends and their long-term health consequences (12). In addition, cross-national inequalities in the burden of tobacco-attributable cancers have not yet been fully quantified. For example, high SDI countries have significantly reduced lung cancer mortality through stringent tobacco-control policies, and low SDI countries continue to experience rising tobacco-attributable cancer mortality due to weak tobacco company marketing strategies and regulations (13). These disparities highlight the need to assess the cancer burden attributable to tobacco use by population and region.

Based on the GBD 2021 database, this study aimed to provide the first systematic assessment of age-standardized disability-adjusted life-years (ASR-DALYs), deaths, and cross-country inequality trends for tobacco-attributable cancers among adults aged 40 and above from 1990 to 2021. By stratifying by region, age, sex, and SDI, we also aimed to reveal the spatial heterogeneity and social determinants of the cancer burden attributable to tobacco and incorporate population growth and aging models to project disease burden trends until 2030. The study could provide insight into developing targeted intervention strategies against cancer.

## 2 Materials and methods

### 2.1 Data sources

Data were obtained from the GBD 2021 and Global Health Data Exchange (GHDx) results. As the most comprehensive global health assessment system currently available, the GBD 2021 provides an extensive assessment of health losses associated with 369 diseases, injuries, and impairments using up-to-date epidemiological data and standardized research methodologies, considering 88 risk factors across 204 countries and territories (14). According to the University of Washington Ethics Review Board, the use of public GBD datasets in this study was exempt from the informed consent process (9). This study strictly followed the normative requirements of the Guidelines for Accurate and Transparent Health Estimates Reporting (GATHER) (15).

The study focused on the global burden of cancer due to tobacco use among middle-aged and older adults (age ≥40 years). Tobacco exposure was defined as a combined exposure pattern involving smoking, chewing tobacco, and exposure to secondhand smoke. Smoking exposure included current and former smokers. Current smokers were defined as individuals who currently smoked tobacco products on a daily or occasional basis. Former smokers were defined as individuals who quit using all tobacco products for at least 6 months. Secondhand smoke exposure was defined as current exposure to secondhand tobacco smoke at home or work. Current chewing tobacco use was defined as current use (use within the last 30 days, where possible, or according to the closest definition available from the survey) of any frequency (any, daily, or less than daily). Chewing tobacco includes local products such as betel quid and tobacco. The malignancies included in the analysis were 16 cancers with clear causes, including malignant tumors of the respiratory system (nasopharyngeal, laryngeal, trachea/bronchus/lung tumors), malignant tumors of the digestive system (esophageal, gastric, colorectal, hepatocellular, and pancreatic tumors), and malignant tumors of the genitourinary system (renal, bladder, prostate, and cervical tumors). The disease burden indicators and age standardization are as follows.

### 2.2 Disease burden indicators and age standardization methods

Disability-adjusted life years (DALYs), the absolute number of deaths, and their corresponding age-standardized rates (ASRs) were used as core indicators. Age standardization was performed to eliminate the interference of population age structure heterogeneity in the intertemporal comparisons. Although the GBD database had provided ASR data for all ages (0–99+ years), to accurately reflect the characteristics of the target population of this study, the research team calculated the ASR for the group aged 40 and above using the direct standardization methods, as following: (1) We systematically identified relevant age groups from the standard population framework, with a focus on those aged 40 and above, using age distribution data from the GBD database. (2) We constructed a new standard population that fit the specific needs of this study, based on the age group proportions of the standard population, aiming to reflect the age distribution of the target population accurately. (3) We used a direct standardization method to calculate the ASRs of the selected age groups. Specifically, the ASR was derived by multiplying the crude rate of each age group by the weight of that group in the newly constructed standard population and summing the weighted values.

### 2.3 Sociodemographic index

The SDI is a composite indicator that covers three dimensions of development: income (Gross Domestic Product (GDP) per capita), education (average and expected years of schooling), and the total fertility rate. The SDI ranged 0–1, with higher SDIs indicating greater socioeconomic development (16). Based on the country-level SDI assessment, the 204 countries and territories in the GBD 2021 were categorized into (low, low-middle, medium, middle-high, and high) quintiles to provide a basis for comparing health outcomes across different levels of socioeconomic development.

### 2.4 Burden description

DALYs, deaths, and their corresponding ASRs were expressed as 95% uncertainty interval (UI) values. To assess the trends in ASR DALYs and ASR deaths from 1990 to 2021, we calculated the estimated annual percentage change (EAPC) (17), which was estimated using a linear regression model designed to describe the trajectory of the ASR over a defined timespan. The formula applied is as follows: y = α + βX + e. Y denotes the natural logarithm of the ASR, X denotes the calendar year, α is the intercept, β denotes the slope or trend, and e denotes the error term. The EAPC is derived from the formula 100 x [exp(β) - 1], which reflects the annual percentage change. A linear regression model was used to determine the 95% confidence interval (CI) associated with EAPC. The average annual percentage change (AAPC), calculated using a segmented linear regression model (e.g., joinpoint regression model), was also used to describe trends in the ASR for DALYs and deaths. Joinpoint regression analyses were used to scrutinize the temporal patterns of cancer burden attributable to tobacco exposure and identify key moments when significant changes occurred. A Bayesian age-period cohort (BAPC) model incorporating an integrated nested Laplace approximation was used to predict future trends (18, 19). The computational process was performed using the R package BAPC. Pyramid plots were used to visualize the distribution of ASRs for DALYs and deaths as well as the absolute number of DALYs and deaths associated with tobacco exposure and cancer for different age groups and sexes. Heat maps were used to depict the geographic distribution of ASRs for DALYs and deaths as well as the number of DALYs and deaths.

### 2.5 Decomposition analysis

To further investigate the potential effects of tobacco exposure on cancer epidemiology from 1990 to 2021, we utilized decomposition analysis to disaggregate the changes in DALYs and deaths due to the risk of tobacco exposure as a result of aging, population growth and epidemiological changes. This approach allowed quantification of the contribution of each factor to the overall change. For this decomposition analysis, we used the method developed by Gupta et al. (20), which summarizes the contributions of different elements to the observed change by mathematically separating the standardized impact of each contributing multiplying factor.

### 2.6 Cross-country inequality analysis

Following World Health Organization guidelines, we used the slope inequality index (SII) and concentration index (CI) to assess differences in cancer due to tobacco exposure across countries with different SDI levels (21). The SII quantifies inequalities in DALYs and mortality across SDI levels in a population. It is calculated by regressing health outcomes (e.g., ASR DALYs or ASR deaths) on the SDI rankings. The slope of the regression line represents SII. Positive slopes indicate that health outcomes improve with socioeconomic status, whereas negative slopes indicate the opposite. The CI was used to assess the concentration of DALYs and deaths in different countries. Lorenz curves were constructed to represent the cumulative distribution of DALYs and deaths in relation to the cumulative proportion of the population sorted by SDI. The CI was calculated for each country by dividing the area between Lorenz curve and line of perfect equivalence by the entire area below line of perfect equivalence. The CI values ranged from -1 to 1. Negative CIs indicate higher concentrations of DALYs or deaths in populations with lower SDIs, whereas positive CIs indicate higher concentrations in populations with higher SDI values.

### 2.7 Statistical analyses

All analyses were performed using the R statistical computing software (version 4.4.1; Posit PBC, Boston, MA, USA) and Stata MP17 (StataCorp LLC, University City, TX, USA). Visualizations were performed using Adobe Illustrator 2023 (version 27.8.1; Adobe Inc., San Jose, CA, USA) and Adobe Photoshop 2023 (version 24.7.0; Adobe Inc.).

## 3 Results

### 3.1 Global trends and demographic stratification of tobacco-attributable cancer burden

**Figure 1:**
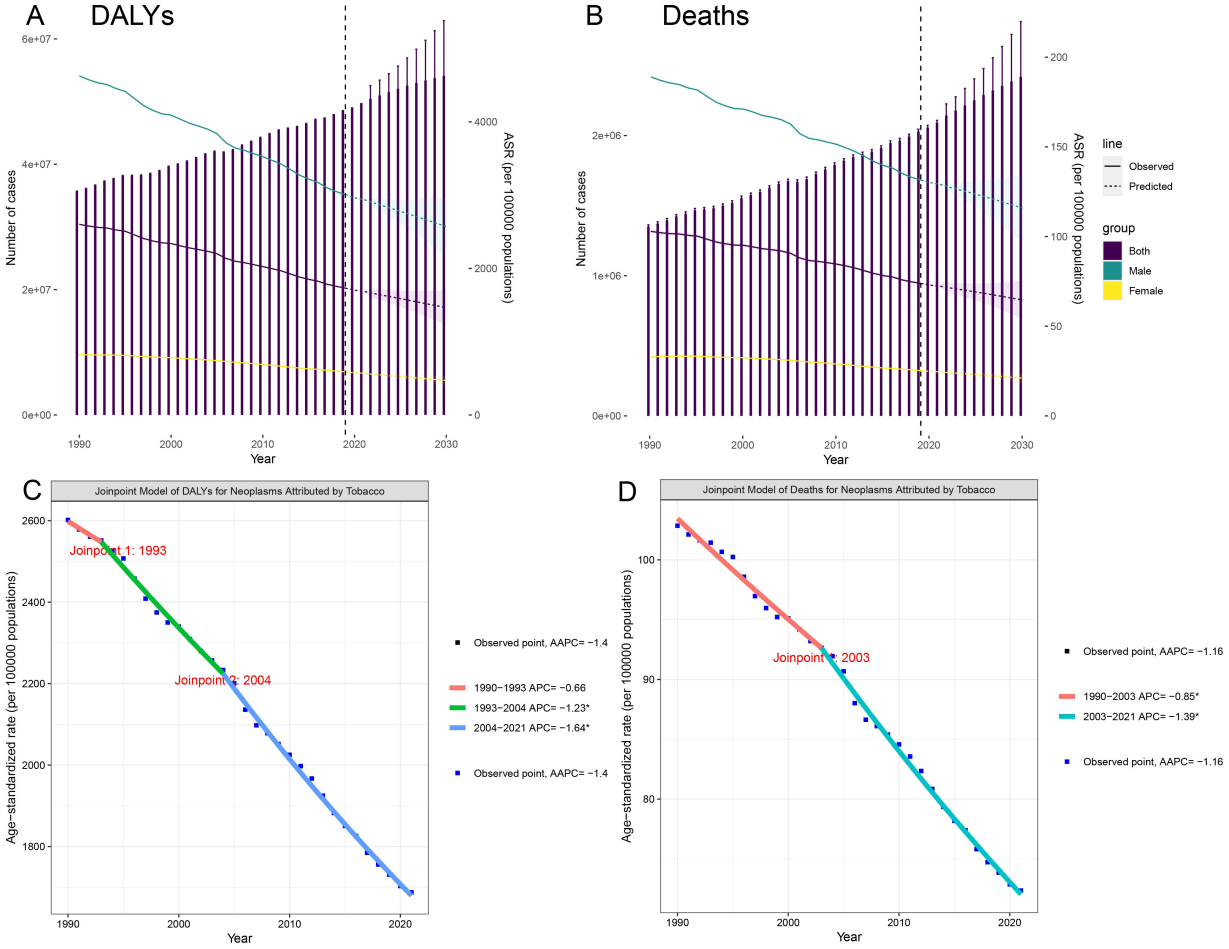
Trends and projections for the global burden of tobacco-attributable cancers in individuals aged 40 and older. **(A)** DALYs. **(B)** Deaths. **(C)** Joinpoint regression analysis of DALYs. **(D)** Joinpoint regression analysis of Deaths.

In 2021, tobacco exposure continued to impose a substantial cancer burden on the global population aged ≥40. ASR DALYs reached 16,874.9 per 100,000 population (95% CI: 13,872.3–20,073.4), accounting for 49,605,504.38 total DALYs (95% UI: 40,809,873.21–58,981,246.61). The EAPC of -1.44 (95% CI: -1.49 to -1.40) demonstrated a sustained global decline in ASR DALYs. Notably, the most pronounced reduction occurred between 2004 and 2021, showing an AAPC of -1.64. The projections suggest a continued 13% decline in the ASR DALYs rate by 2030, reaching approximately 14,646.8 per 100,000 people (**Table 1**, **Figure 1**).

**Table 1:** Global, age- and sex-specific DALYs cases and ASR DALYs for age ≥40 years tobacco-attributable cancers in 2021, changing trends from 1990 to 2021, and prediction of 2030.

Variable	2021		1990-2021
	DALYs cases	ASR-DALYs per 100,000 (95% UI)	EAPC of DALYs
Global	49605504.38 (40809873.21 to 58981246.61)	1687.49 (1387.23 to 2007.34)	-1.44 (-1.49 to -1.4)
Global Prediction of 2030	54103566.08 (45309782.90 to 62897349.25)	1464.68 (1225.78 to 1703.58)	
Sex			
Male	40807852.43 (33959710.73 to 48527347.14)	2940.18 (2444.39 to 3497.46)	-1.51 (-1.55 to -1.47)
Male Prediction of 2030	44868541.16 (37431595.22 to 52305487.10)	2570.91 (2169.14 to 2972.68)	
Female	8797651.94 (6514408.08 to 11306011.15)	570.43 (421.77 to 733.6)	-1.24 (-1.33 to -1.15)
Female Prediction of 2030	9235024.92 (7878187.69 to 10591862.15)	471.58 (406.4 to 536.75)	
Age (years)			
40–44 years	1364344.75 (1054301.41 to 1675895.61)	272.73 (210.75 to 335.01)	-2.79 (-2.92 to -2.66)
45–49 years	2647161.85 (2085578.3 to 3231183.09)	559.06 (440.46 to 682.4)	-2.39 (-2.55 to -2.22)
50–54 years	5005045.64 (4011524.82 to 6064714.84)	1124.92 (901.62 to 1363.09)	-2.15 (-2.28 to -2.02)
55–59 years	7305577.74 (6018660.03 to 8697283.64)	1846.11 (1520.91 to 2197.79)	-1.74 (-1.81 to -1.67)
60–64 years	8093769.62 (6809320.75 to 9481754.8)	2528.92 (2127.59 to 2962.61)	-1.51 (-1.58 to -1.43)
65–69 years	8902917.54 (7498653.33 to 10426678.35)	3227.54 (2718.46 to 3779.94)	-1.36 (-1.42 to -1.3)
70–74 years	7469578.29 (6185301.29 to 8816845.31)	3628.84 (3004.92 to 4283.37)	-1.08 (-1.17 to -0.99)
75–79 years	4556241.65 (3740878.89 to 5431513.09)	3454.72 (2836.48 to 4118.39)	-0.64 (-0.76 to -0.53)
80–84 years	2502321.59 (2030905.25 to 3003016.15)	2857.08 (2318.83 to 3428.76)	-0.23 (-0.35 to -0.1)
85–89 years	1249881.52 (998891.24 to 1513813.5)	2733.67 (2184.72 to 3310.92)	0.17 (0.05 to 0.29)
90–94 years	415795.47 (313416.95 to 517706.43)	2324.26 (1751.97 to 2893.93)	0.13 (0.07 to 0.19)
95+ years	92868.72 (62440.96 to 120841.82)	1703.92 (1145.64 to 2217.16)	-0.03 (-0.13 to 0.07)

ASR mortality rates were 72.36 per 100,000 (95% CI: 59.40–86.09), corresponding to 2,092,020.63 deaths (95% UI: 1,719,655.29–2,487,163.10). Although mortality trends diverged from DALY patterns by reaching an inflection point in 2003, the post-2003 period witnessed accelerated declines (AAPC: -1.39). Current models predict that the mortality rate will decrease to 64.59 per 100,000 individuals by 2030 (**Supplementary Table S1**, **Figure 1**). Notably, both sexes were projected to experience mortality rate increases through 2021 (95% CI: 59.4–86.09).

In 2021, the DALYs for males and females were 40.8 million and 8.79 million, respectively, with 1.7 million and 380,000 deaths. Gender differences in disability-adjusted life years and deaths reached 32.01 million and 1.32 million, respectively. Worldwide, men aged 40 and above exhibited significantly higher DALY and mortality rates than women. During the study period, both male and female ASR DALYs and ASR deaths decreased; it is expected that by 2030, the decline in female patients will be greater than that in male patients (**Table 1**, **Supplementary Table S1**).

In 2021, DALYs and deaths initially increased with age and then decreased, reaching their peaks in the 65–69 and 70–74 age groups, respectively. From 1990 to 2021, ASR-DALYs and ASR-deaths decreased in most age groups. Yet there was an increase in DALYs in the 85–89 and 90–94 age groups and an increase in deaths in the 85–89, 90–94, and 95+ age groups, indicating an increasing trend in the cancer burden caused by smoking among the older population aged 85 and above (**Table 1**, **Supplementary Table S1**).

### 3.2 Tobacco-attributable cancer burden by exposure routes and cancer types

**Figure 2:**
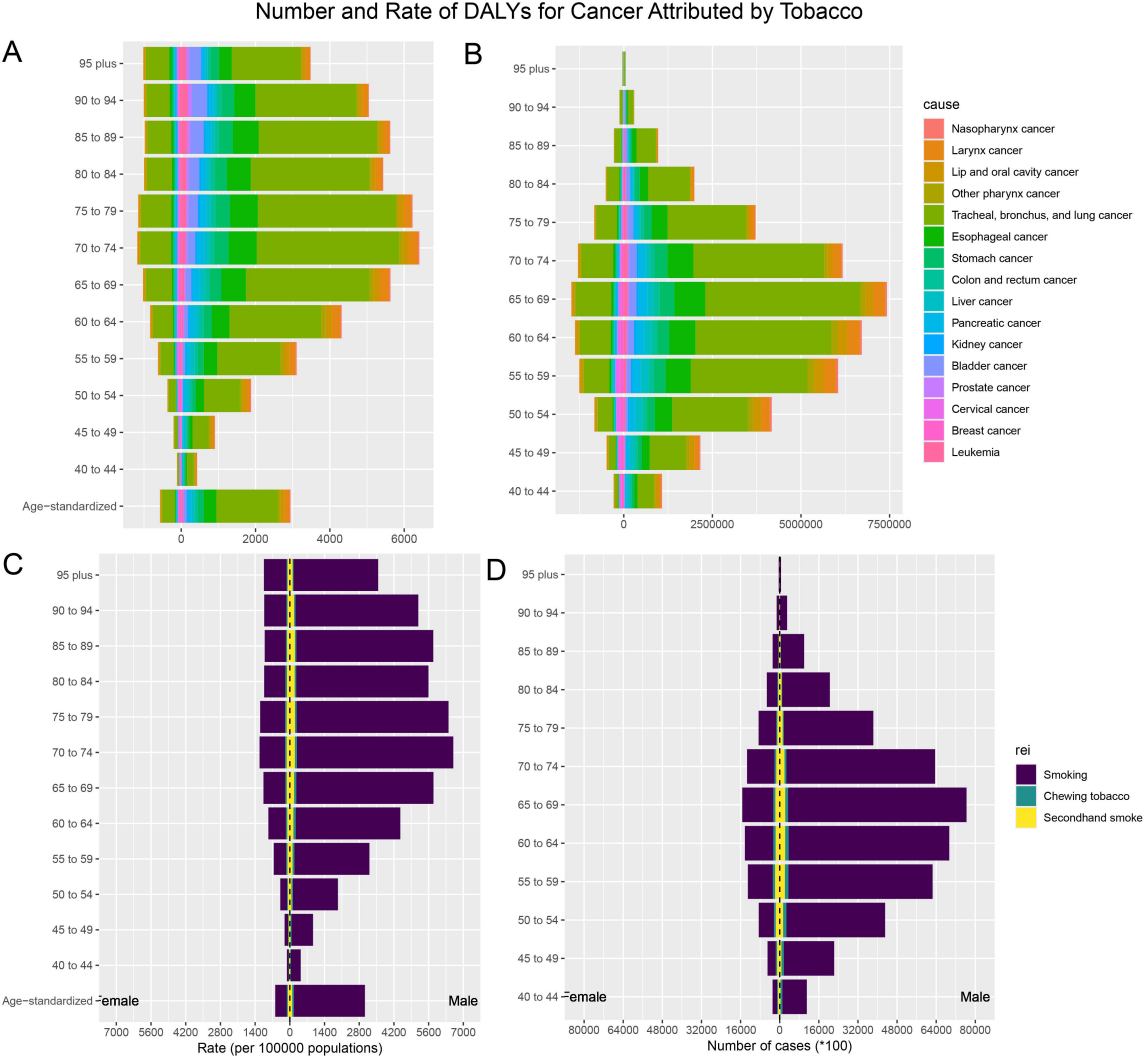
The burden of tobacco-attributable cancer and cancer stratified by age and sex. **(A)** ASR-DALYs for cancers attributable to tobacco. **(B)** Number of DALYs cases for cancers attributable to tobacco. **(C)** ASR-DALYs for tobacco-attributable cancer. **(D)** Number of DALYs cases for tobacco-attributable cancer.

Smoking, chewing tobacco, and secondhand smoke were the most significant contributors to ASR DALYs. Tobacco smoking was the highest at 1,603.98 (95% UI: 1,336.9–1,895.27) per 100,000 people, highlighting its huge public health burden. ASR-DALYs for secondhand smoke and chewing tobacco were 85.49 (95% UI: 7.83–165.07) per 100,000 and 50.52 (95% UI: 38.16–64.27) per 100,000, respectively. From 1990 to 2021, smoking, chewing tobacco, and secondhand smoke showed reductions (downward trends) in ASR DALYs. The EAPC was -1.49 (95% UI: -1.54 to -1.44) for smoking, -1.21 (95% UI: -1.27 to -1.15) for secondhand smoke, and -0.16 (95% UI: -0.24 to -0.08) for chewing tobacco (**Table 2**). The ASR DALYs and ASR deaths attributable to the risk of tobacco exposure varied by sex and age. Smoking was the most prominent tobacco burden, and its impact first increased with age, with the highest rate of DALYs in the group of 70–74 years old, and then decreased with age, especially in the male population. Among all age groups, this rate was significantly lower in females than males (**Figures 2**, **Supplementary Figure S1**).

**Table 2:** Tobacco type and cancer type specific DALYs cases and ASR DALYs for age ≥40 years tobacco-attributable cancers in 2021, changing trends from 1990 to 2021, and prediction of 2030.

Variable	2021		1990-2021
	DALYs cases	ASR-DALYs per 100,000 (95% UI)	EAPC of DALYs
Carcinogen risk			
Smoking	47159120.67 (39337262.79 to 55704244.39)	1603.98 (1336.9 to 1895.27)	-1.49 (-1.54 to -1.44)
Chewing tobacco	1483139.53 (1120437.7 to 1886550.78)	50.52 (38.16 to 64.27)	-0.16 (-0.24 to -0.08)
Secondhand smoke	2508916.42 (229923.92 to 4840717.59)	85.49 (7.83 to 165.07)	-1.21 (-1.27 to -1.15)
Cancer			
Nasopharynx cancer	368589.44 (266113.12 to 483865.27)	12.47 (9.01 to 16.38)	-2.48 (-2.68 to -2.29)
Larynx cancer	2030941.46 (1799344.96 to 2276996.88)	68.75 (60.86 to 77.1)	-2.25 (-2.32 to -2.17)
Lip and oral cavity cancer	2152627.29 (1709677.89 to 2606679.1)	73.17 (58.08 to 88.63)	-0.64 (-0.68 to -0.59)
Other pharynx cancer	1013152.06 (797227.28 to 1222354.31)	34.18 (26.89 to 41.27)	-0.87 (-0.94 to -0.81)
Tracheal, bronchus, and lung cancer	28390385.73 (24466230.11 to 32764511.24)	965.45 (831.41 to 1114.55)	-1.25 (-1.32 to -1.18)
Esophageal cancer	5080668.73 (3942911.76 to 6379169.95)	172.72 (134.02 to 216.84)	-1.66 (-1.77 to -1.55)
Stomach cancer	2478088.21 (1910632.82 to 3227935.64)	84.48 (65.11 to 109.99)	-2.9 (-2.94 to -2.85)
Colon and rectum cancer	1188136.36 (741166.52 to 1664939.58)	40.45 (25.22 to 56.72)	-1.26 (-1.28 to -1.23)
Liver cancer	1404063.23 (472513.34 to 2367315.11)	47.63 (16.02 to 80.35)	-1.01 (-1.12 to -0.91)
Pancreatic cancer	1744663.99 (1504475.8 to 2010783.62)	59.24 (51.04 to 68.34)	-0.6 (-0.63 to -0.56)
Kidney cancer	379883.31 (231243.85 to 538168.6)	12.95 (7.86 to 18.38)	-1.14 (-1.26 to -1.02)
Bladder cancer	1227679.35 (1023220 to 1475972.62)	42.43 (35.3 to 51.07)	-1.85 (-1.91 to -1.79)
Prostate cancer	280762.94 (127894.98 to 459130.54)	9.7 (4.41 to 15.92)	-2.07 (-2.16 to -1.98)
Cervical cancer	654242.57 (383945.99 to 968759.19)	22.24 (13.05 to 32.93)	-2.42 (-2.45 to -2.38)
Breast cancer	512279.6 (174571.79 to 860569.29)	17.46 (5.93 to 29.36)	-1.78 (-1.85 to -1.72)
Leukemia	699340.1 (251241.21 to 1184137.89)	24.16 (8.64 to 41.03)	-1.72 (-1.8 to -1.64)

Among the 16 cancers included in this study, tracheal, bronchial, and lung cancers had significantly higher ASR-DALYs and death rates than other types of cancers. The ASR-DALYs for tracheal, bronchial, and lung cancers was 965.45 (95% UI: 831.41–1,114.55) per 100,000 people, which was 5.6 times higher than that of the second-ranked esophageal cancer, which had an ASR-DALY of 172.72 (95% UI: 134.02–216.84) per 100,000 people. The ASR deaths were 42.6 per 100,000 (95% UI: 36.48–49.25) for tracheal, bronchial, and lung cancers and 7.54 per 100,000 (95% UI: 5.82–9.47) for esophageal cancers. Tracheal, bronchial, and lung cancers showed a clear male predominance (**Table 2**, **Supplementary Table S1**, **Figures 2**, **Supplementary Figure S1**).

### 3.3 Tobacco-attributable cancer burden stratified by SDI

The global cancer burden attributable to tobacco exposure showed significant regional differences that were closely associated with the SDI levels. In 2021, the highest ASR DALYs were found in the medium-high SDI regions at 2,426.26 (95% UI: 1,978.43–2,947.34) per 100,000 people, while the lowest ASR-DALYs were found in the low SDI regions at 551.12 (95% UI: 426.03–686.25). From 1990 to 2021, all regions showed a decreasing trend, with high SDI regions showing the largest decrease in EAPC at -2.06 (95% CI: -2.15 to -1.97) and low and medium SDI regions showing the smallest decrease, with an EAPC of -0.6 (95% CI: -0.62 to -0.57). Similarly, ASR mortality rates showed regional variation. The highest number of ASR deaths was found in the middle-SDI and high-SDI regions, whereas the lowest was found in the low-SDI regions. Similarly, from 1990 to 2021, high SDI regions had the largest decline in EAPC at -1.8 (95% CI: -1.88 to -1.72) (**Table 3**, **Supplementary Table S1**).

**Table 3:** SDI and GBD region specific DALYs cases and ASR DALYs for age ≥40 years tobacco-attributable cancers in 2021, changing trends from 1990 to 2021, and prediction of 2030.

Variable	2021		1990-2021
	DALYs cases	ASR-DALYs per 100,000 (95% UI)	EAPC of DALYs
SDI			
High	11652453.33 (9716491.95 to 13559684.89)	1753.07 (1465.87 to 2035.24)	-2.06 (-2.15 to -1.97)
High-middle	16354416.28 (13357508.24 to 19849757.79)	2426.26 (1978.43 to 2947.34)	-1.23 (-1.3 to -1.16)
Middle	15566523.03 (12253828.83 to 19332588.84)	1665.11 (1310.86 to 2067.06)	-1.08 (-1.12 to -1.04)
Low-middle	4988098.14 (4090967.67 to 5895540.74)	988.08 (811.48 to 1167.01)	-0.6 (-0.62 to -0.57)
Low	994395.32 (765163.16 to 1243048.09)	551.12 (426.03 to 686.25)	-1.03 (-1.1 to -0.96)
GBD region			
High-income	11476000.28 (9551666.75 to 13370336.79)	1683.67 (1407.49 to 1954.85)	-2.11 (-2.19 to -2.02)
Southeast Asia, East Asia, and Oceania	24192049.71 (18742820.41 to 30696137.38)	2436.22 (1887.33 to 3087.57)	-0.73 (-0.81 to -0.64)
South Asia	4711100.41 (3810186.56 to 5662553)	907.18 (734.37 to 1089.84)	-0.96 (-1.03 to -0.89)
Central Europe, Eastern Europe, and Central Asia	4667869.77 (3969840.9 to 5327410.21)	2162.68 (1836.07 to 2470.65)	-1.65 (-1.76 to -1.54)
North Africa and Middle East	1965729.05 (1552125.58 to 2405208.5)	1233.86 (973.55 to 1511.53)	-0.91 (-1.01 to -0.82)
Latin America and Caribbean	1758915.67 (1412706.49 to 2116988.42)	830.77 (666.85 to 1000.22)	-2.25 (-2.34 to -2.16)
Sub-Saharan Africa	833839.48 (621051.7 to 1054975.14)	476.79 (357.29 to 601.48)	-1.04 (-1.17 to -0.9)

### 1.1 Tobacco-attributable cancer burden in GBD super-regions

**Figure 3:**
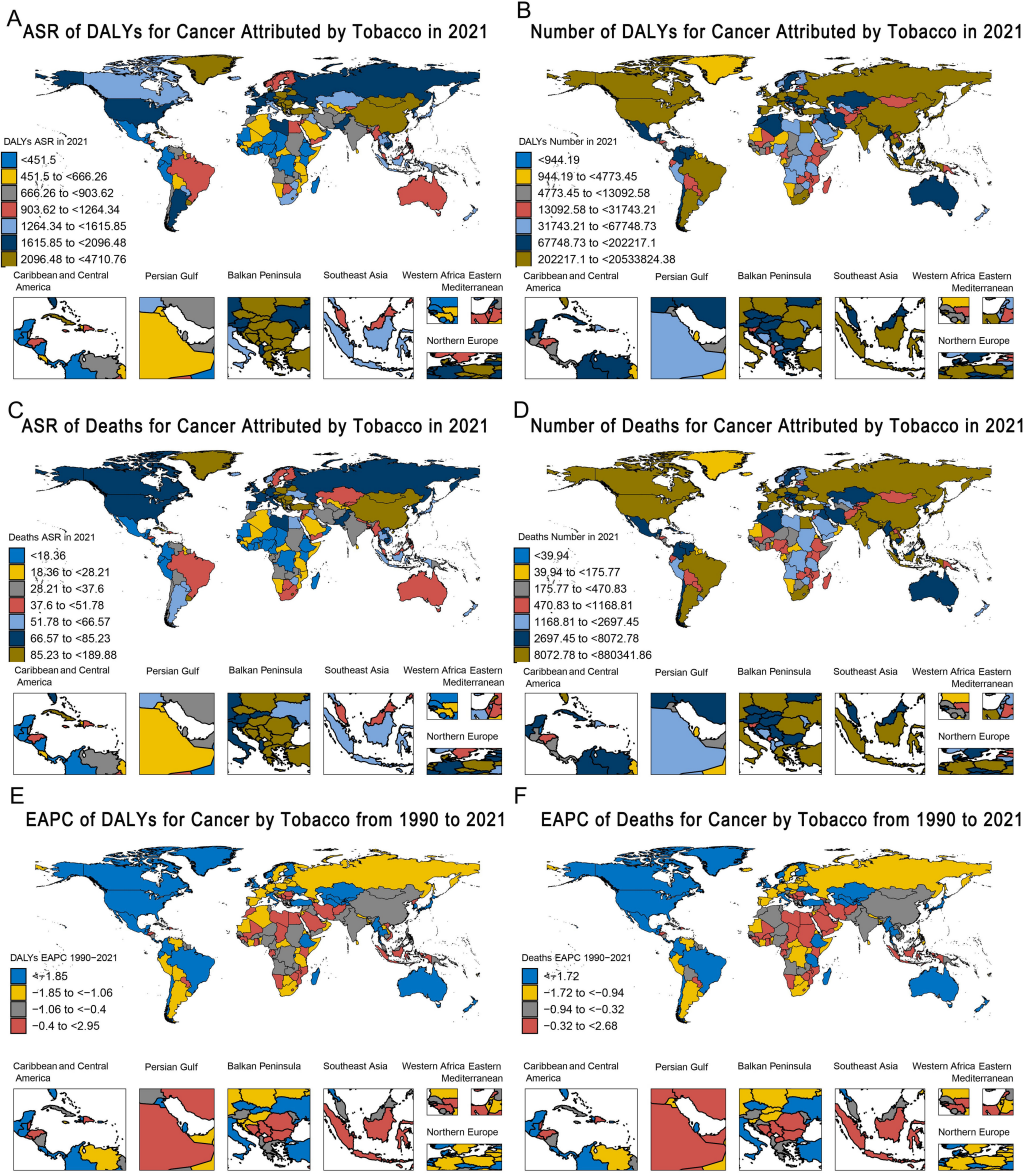
Global heatmap of the burden of tobacco-attributable cancer in 2021 and changing trends from 1990–2021. **(A)** ASR of DALYs. **(B)** Number of DALYs. **(C)** ASR of deaths. **(D)** Number of Deaths. **(E)** EAPC of DALYs. **(F)** EAPC of Deaths.

Among the seven GBD super-regions, Southeast Asia, East Asia, and Oceania had the highest cancer burden attributable to tobacco exposure among people aged 40 and above, with ASR-DALYs of 2,436.22/100,000 population in 2021. This was followed by Central and Eastern Europe and Central Asia (ASIR: 2,162.68 per 100,000 people) and high-income regions (ASIR: 1,683.67 per 1 million population) (**Table 3**). From 1990 to 2021, ASR DALYs show a decreasing trend in all seven GBD regions, with the highest decreasing trend in Latin America and the Caribbean (EAPC: -2.25, 95% CI: -2.34 to -2.16) (**Table 3**, **Figure 3**). Similarly, Southeast Asia, East Asia, and Oceania have the highest burden of cancer deaths caused by tobacco exposure, with the highest ASR-death (108.08 per 100,000 population in 2021), followed by Central and Eastern Europe and Central Asia (81.25 per 100,000 population), and high-income regions (72.32 per 100,000 population). From 1990 to 2021, ASR deaths in the seven GBD regions similarly showed a decreasing trend, with the highest decreasing trend in Latin America and the Caribbean (EAPC: -2.12, 95% CI: -2.2 to -2.04) (**Supplementary Table S1**, **Figures 3**, **Supplementary Figure S2**).

**Supplementary Figures S3–S21** provide a comprehensive overview of the global picture of different types of tobacco use and different cancers attributable to tobacco exposure in 2021 as measured by ASR DALYs and ASR deaths. Based on the ASR, several key observations regarding tobacco exposure and cancer burden were highlighted. It is clear that the burden of cancer due to tobacco exposure in 2021 was the highest in North America, Eastern Europe, Central Europe, and parts of Latin America (**Supplementary Figure S3**), while Southern North America and parts of Sub-Saharan Africa reported the highest burden of chewing tobacco exposure (**Supplementary Figure S4**), and the cancer burden attributable to secondhand smoke was particularly high in Eastern Europe, Central Europe, and Southeast Asia (**Supplementary Figure S5**). In addition, the burdens of tracheal, bronchial, and lung cancers were highest in North America, Eastern Europe, and Central Europe in 2021 (**Supplementary Figure S10**).

### 1.2 Decomposition analysis

**Figure 4:**
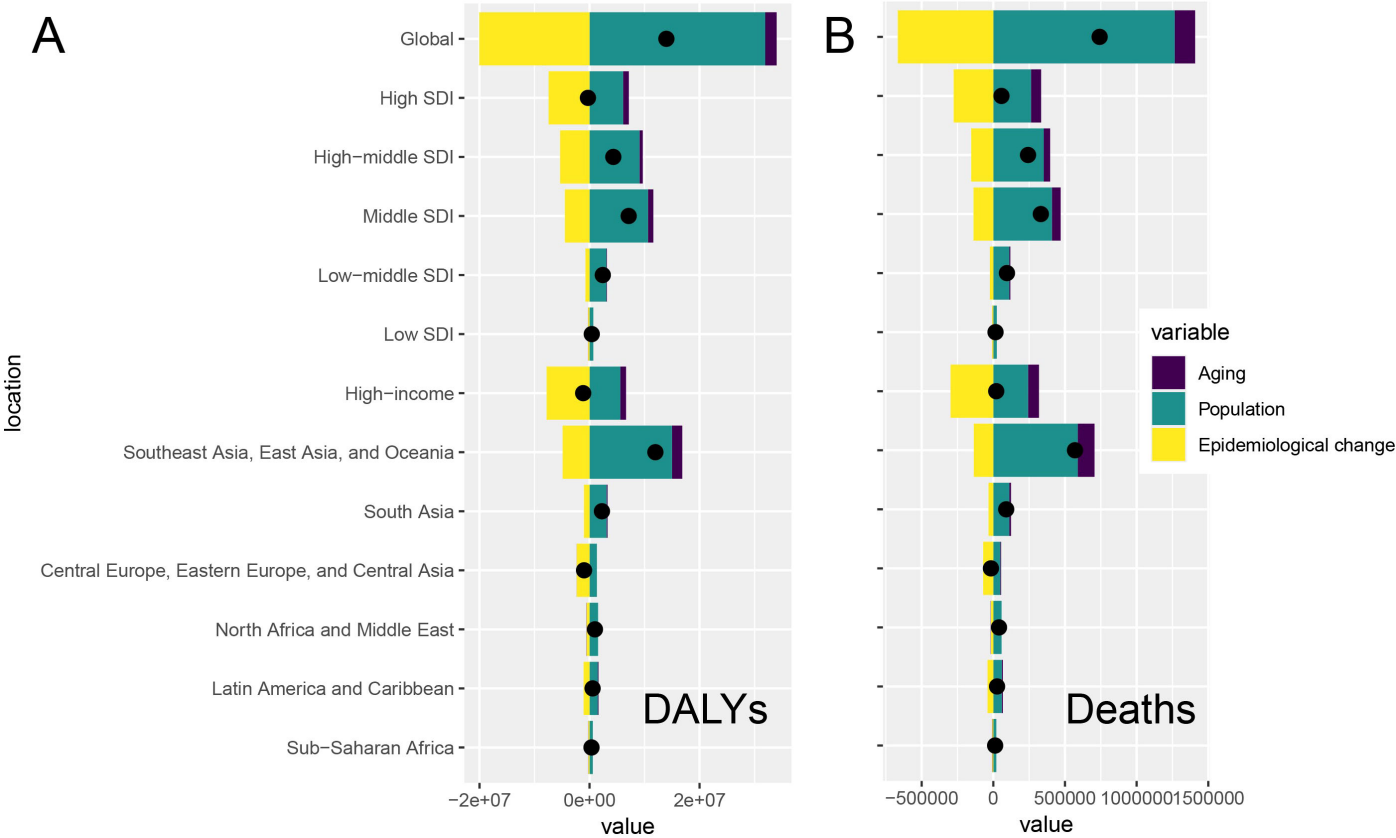
Global and regional decomposition analysis of the impact of tobacco-attributable cancer epidemiology on **(A)** DALYs and **(B)** deaths.

Through decomposition analyses of the original DALYs and deaths from tobacco-attributable cancers, we assessed the impact of tobacco-associated cancers on aging, population growth, and epidemiological changes from 1990 to 2021. Globally, population growth was the leading cause of DALYs and deaths, followed by epidemiological changes; population aging had a relatively minor impact. Population growth was the main driver of DALYs and deaths in most regions, but epidemiological change was the main driver of DALYs and deaths in high-SDI regions. These characteristics were also observed in the seven GBD super regions, with epidemiological changes being the main driver of DALYs and deaths in high-income regions, Eastern and Central Europe, and Central Asia. The impact of epidemiological changes on DALYs and deaths was even greater because of the implementation of superior healthcare and disease prevention strategies in these regions, which were significant in regions with a higher SDI and income (**Supplementary Table S2**, **Figure 4**).

### 1.3 Health inequalities analysis

**Figure 5:**
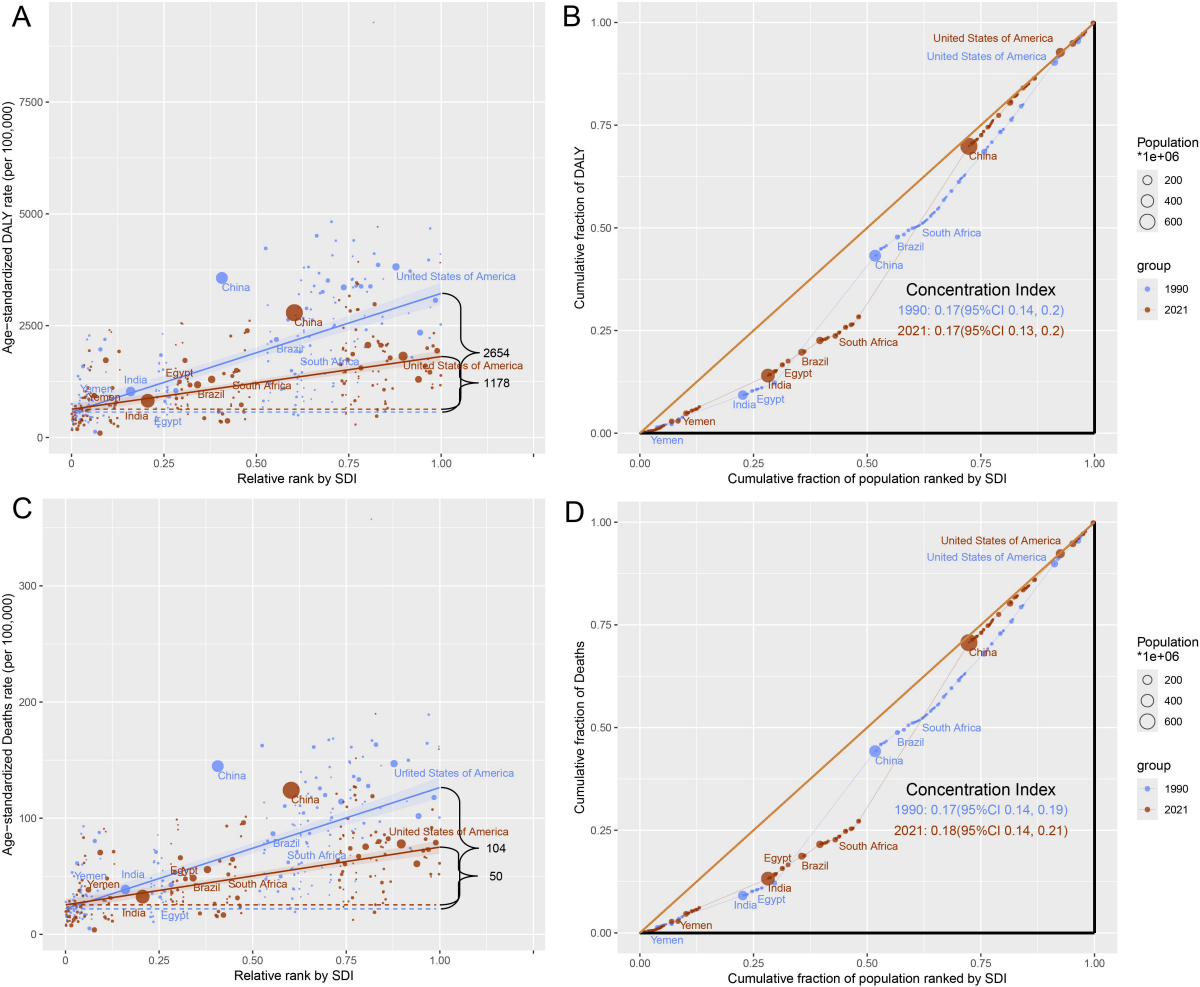
Health inequality of tobacco-attributable cancer in 1990 and 2021. **(A)** SII for DALYs. **(B)** CI for DALYs. **(C)** SII for deaths. **(D)** CI for deaths.

Global health inequalities in the cancer burden due to tobacco exposure showed significant dynamics between 1990 and 2021. The SII of DALYs declined from 2,654/100,000 in 1990 to 1,178/100,000 in 2021, a decrease of 55.6%, suggesting that the difference in ASR DALYs between high- and low-SDI countries has narrowed significantly. The SII for mortality also declined from 104/100,000 to 50/100,000 (a 51.9% decline), further validating the trend of easing inequality. Concentration index (CI) analysis showed no significant change in the equity of the global distribution of DALYs between 1990 and 2021 (CI: 0.17), but the mortality CI edged up from 0.17 (95% CI: 0.14–0.19) to 0.18 (95% CI: 0.14–0.21), suggesting a slight increase in the concentration of death burden in high SDI regions (**Figure 5**, **Supplementary Tables S4**, **S5**).

## 2 Discussion

For tobacco-attributable cancers, ASR-DALYs and ASR-deaths among people aged 40 and above have shown a continuous downward trend globally from 1990 to 2021 and are projected to decrease by 13% by 2030. This trend may be attributed to comprehensive tobacco control policies implemented in multiple countries. For example, the WHO FCTC has significantly reduced smoking prevalence since it was put into force in 2005 by promoting tax increases, public place smoking bans, and tobacco package warning policies (22, 23). High-income countries have led to behavioral changes through nicotine replacement therapy and e-cigarette regulations, such as the United Kingdom Stop Smoking Service, which reduced the smoking prevalence from 27% in 2000 to 14% in 2020 (22). In addition, lung cancer screening programs (e.g., low-dose Computed Tomography (CT) scans in the United States) have increased the rate of early diagnosis and reduced mortality from advanced cancers (23, 24). However, low-income countries with lagging policy implementation may not fully benefit from these measures (2).

DALYs and deaths were significantly higher in men than in women, consistent with the longstanding historical pattern of higher smoking prevalence than in women globally (11, 25). For example, the prevalence of smoking in China is 52.1% in men and 2.7% in women, leading to significant sex differences in lung cancer (26). The greater reduction in the burden on women may be due to targeted interventions (e.g., education on smoking during pregnancy) (27). However, rising female smoking prevalence in regions such as Eastern Europe may offset this trend in the future (23).

In 2021, ASR DALYs and ASR mortality rates peaked at 65–69 and 70–74 years old and then decreased, consistent with the long-term incubation period for tobacco-attributable cancers (28, 29). Cancers caused by smoking, such as lung cancer, typically manifest 20–30 years after exposure, thereby increasing the burden on the older population. Smith et al. showed that the incidence of lung cancer peaks at the age of 70–74 years old, which is consistent with the cumulative effect of smoking (30, 31). This study also found that from 1990 to 2021, ASR-DALYs and ASR deaths in most age groups showed a decreasing trend. However, DALYs increased in the 85–89 and 90–94 age groups, and deaths increased in the 85–89, 90–94, and 95+age groups, indicating an increased burden of tobacco-attributable cancer in the population over 85 years of age. This may be because the smoking rate in the mid-20th century was significantly higher than that in recent years, and the older population may have had a higher cumulative smoking exposure when they were young, leading to an increased risk of cancer. Meza analyzed the specific smoking history of birth cohorts from 1965 to 2009 and found that early birth cohorts (such as 1920–1940) had higher smoking rates and longer smoking durations (32).

The tobacco-attributable cancer burden was strongly correlated with SDI levels, with the highest 2021 ASR-DALYs in medium-SDI to high-SDI areas (2,426.26/100,000) and the lowest in low-SDI areas (551.12/100,000). A higher historical smoking prevalence and diagnostic capacity in areas with a high SDI, coupled with an aging population, may lead to a higher burden (23, 33), a pattern confirmed by the GBD 2019 study (11). Inadequate cancer registry systems in low-SDI regions may lead to an underestimation of the burden. For example, sub-Saharan Africa has less than 30% coverage of cancer registries, and cancer deaths in rural India are often categorized as ‘non-specific causes’ (34). In addition, lack of healthcare resources leads to delays in diagnosis, which further masks the true burden (27). Southeast Asia, East Asia, and Oceania had the highest burden (2,436.22/100,000), which may be related to the culture of smoking and exposure to secondhand smoke. High SDI regions showed the greatest reduction in burden from 1990 to 2021, which may be attributed to the effective implementation of tobacco control policies (e.g., FCTC) (22). Latin America and the Caribbean showed the largest decreases, which may be related to regional tobacco control initiatives and higher tobacco taxes (23, 35).

Smoking was the largest contributor to ASR-DALYs (1603.98/100,000), far exceeding those of secondhand smoke (85.49/100,000) and chewing tobacco (50.52/100,000), underscoring its public health impact. The WHO (2021) report (36) states that tobacco use is the leading cause of cancer-related deaths globally and is particularly associated with cancers of the trachea, bronchus, and lungs. The high burden of chewing tobacco in Southeast Asia (e.g., betel-tobacco mixtures in India) requires targeted interventions (37). The declining trend in ASR-DALYs for the three types of tobacco exposures from 1990 to 2021 may be related to global tobacco control policies and health education outreach (22, 38).

Decomposition analyses revealed that population growth was the main driver of DALYs and deaths globally, followed by epidemiological changes, with aging having the lowest impact. High SDI regions, on the contrary, are dominated by epidemiological changes, which may be due to lifestyle shifts (e.g., reduced smoking) and medical advances that reduce morbidity (23). High-income and Eastern European regions showed similar trends, with superior medical care likely amplifying the impact of epidemiological changes. However, a study by Bray et al. (39) also showed that population aging drives the cancer burden, especially in low SDI regions. This result suggests that regional differences require targeted policies, such as controlling the population burden in low-SDI regions and optimizing prevention strategies in high-SDI regions.

From 1990 to 2021, the SII of DALYs decreased from 2,654/100000 to 1,178/100000 (a decrease of 55.6%), and the SII of deaths decreased from 104/100000 to 50/100000 (a decrease of 51.9%), indicating a reduction in health inequality. The WHO (2019) report (40) shows that the implementation of the FCTC significantly reduces smoking rates and narrows the gap between countries. However, the CI of deaths increased slightly (0.17–0.18), indicating a slight increase in the burden of death in low-SDI areas, possibly due to insufficient medical resources (34). For example, only 28% of cancer deaths in sub-Saharan Africa are accurately registered, and there is a lack of radiotherapy facilities and targeted drug supply, resulting in a cancer mortality rate of up to 75%, whereas high-income countries account for only 46% (41). However, the popularity of e-cigarettes in high-SDI countries may reshape future patterns of inequality. Although the situation of health inequality between countries has eased, it still exists, indicating that although countries with high SDI have implemented certain tobacco control measures, more targeted strategies still need to be developed to mitigate the impact of tobacco-attributable cancer. Countries with medium SDI should strengthen their tobacco control policies and increase related resource investments. Due to insufficient medical resources, countries with low SDI urgently need to improve their cancer diagnosis and treatment levels to meet the growing demand for health.

This study has few limitations. First, there are data quality limitations of the GBD; self-reported smoking is susceptible to social desirability bias, and some studies have shown that the error rate of coding cancer causes of death in less developed regions can be as high as 40% (34). Second, exposure assessment may be biased, with exposure to secondhand smoke relying on model estimation and lacking individualized biomarker validation (22). Finally, lagged effects may not be adequately captured, and the long-term latent nature of tobacco carcinogenesis may lead to an underestimation of the future impact of new tobacco products (e.g., e-cigarettes) (27).

## 3 Conclusions

This study showed that the burden of tobacco-induced cancers among people aged 40 and above has been declining since 1990, and cross-national inequalities in the burden of tobacco-attributable cancers between countries and regions due to differences in social development have decreased but still exist. To reduce health inequalities and achieve universal health coverage, we need to pay more attention to tobacco use among middle-aged and older populations, implement effective public health measures. High SDI countries need to continue optimizing public health interventions to reduce the burden of tobacco-attributable cancer. Medium SDI countries should also gradually increase public health investment in tobacco control. Low SDI countries may need to improve their cancer diagnosis and treatment capabilities.

## Data Availability

Publicly available datasets were analyzed in this study. This data can be found here: the Global Health Data Exchange (http://ghdx.healthdata.org/gbd-results-tool).

## References

[1] Global Burden of Disease Collaborative Network. Global Burden of Disease Study. (2022). Avaible at: https://www.healthdata.org/research-analysis/gbd (Accessed March 5, 2025).

[2] Bray F Laversanne M Sung H Ferlay J Siegel RL Soerjomataram I. Global cancer statistics 2022: GLOBOCAN estimates of incidence and mortality worldwide for 36 cancers in 185 countries. CA: Cancer J Clin. (2024) 74:229–63. doi: 10.3322/caac.21834 38572751

[3] Chen S Cao Z Prettner K Kuhn M Yang J Jiao L. Estimates and projections of the global economic cost of 29 cancers in 204 countries and territories from 2020 to 2050. JAMA Oncol. (2023) 9:465–72. doi: 10.1001/jamaoncol.2022.7826, PMID: 36821107 PMC9951101

[4] World Health Organization. Cancer Prevention and Control in the Context of an Integrated Approach. WHO Technical Report Series. (2017). Available at: https://www.who.int/publications/i/item/cancer-prevention-and-control-in-the-context-of-an-integrated-approach (Accessed March 5, 2025).

[5] International Agency for Research on Cancer (IARC). IARC monographs on the evaluation of carcinogenic risks to humans - Volume 83: Tobacco smoke and involuntary smoking, World Health Organization, IARC. (2004). PMC4781536 15285078

[6] Nethan ST Sinha DN Kedar A Kumar V Sharma S Hariprasad R. Tobacco use among urban slum dwellers attending a cancer screening clinic in the National Capital Region of India: a cross-sectional study. Ecancermedicalscience. (2021) 15:1230. doi: 10.3332/ecancer.2021.1230, PMID: 34158834 PMC8183651

[7] Eriksen M MJ Schluger N. The Tobacco Atlas. 6th ed. Atlanta, GA: American Cancer Society (2022).

[8] Program. NT. Tobacco-Related Exposures, Report on Carcinogens, Fifteenth Edition. Triangle Park, NC: National Institute of Environmental Health and Safety (2021).

[9] GBD 2019 Risk Factors Collaborators. Global burden of 87 risk factors in 204 countries and territories, 1990-2019: a systematic analysis for the Global Burden of Disease Study 2019. Lancet (London England). (2020) 396:1223–49. doi: 10.1016/S0140-6736(20)30752-2, PMID: 33069327 PMC7566194

[10] Jha P Peto R. Global effects of smoking, of quitting, and of taxing tobacco. New Engl J Med. (2014) 370:60–8. doi: 10.1056/NEJMra1308383, PMID: 24382066

[11] Safiri S Nejadghaderi SA Abdollahi M Carson-Chahhoud K Kaufman JS Bragazzi NL. Global, regional, and national burden of cancers attributable to tobacco smoking in 204 countries and territories, 1990-2019. Cancer Med. (2022) 11:2662–78. doi: 10.1002/cam4.4647, PMID: 35621231 PMC9249976

[12] GBD 2021 Risk Factors Collaborators. Global burden and strength of evidence for 88 risk factors in 204 countries and 811 subnational locations, 1990-2021: a systematic analysis for the Global Burden of Disease Study 2021. Lancet (London England). (2024) 403:2162–203. doi: 10.1016/S0140-6736(24)00933-4, PMID: 38762324 PMC11120204

[13] Zhang Z Zhao Y Bian Y. A role of socioeconomic status in cognitive impairment among older adults in Macau: A decomposition approach. Front Aging Neurosci. (2022) 14:804307. doi: 10.3389/fnagi.2022.804307, PMID: 35211006 PMC8862725

[14] GBD 2019 Diseases and Injuries Collaborators. Global burden of 369 diseases and injuries in 204 countries and territories, 1990-2019: a systematic analysis for the Global Burden of Disease Study 2019. Lancet (London England). (2020) 396:1204–22. doi: 10.1016/S0140-6736(20)30925-9, PMID: 33069326 PMC7567026

[15] Stevens GA Alkema L Black RE Boerma JT Collins GS Ezzati M. Guidelines for accurate and transparent health estimates reporting: the GATHER statement. PloS Med. (2016) 13:e1002056. doi: 10.1016/S0140-6736(16)30388-9, PMID: 27351744 PMC4924581

[16] GBD 2019 Stroke Collaborators. Global, regional, and national burden of stroke and its risk factors, 1990-2019: a systematic analysis for the Global Burden of Disease Study 2019. Lancet Neurol. (2021) 20:795–820. doi: 10.1016/S1474-4422(21)00252-0, PMID: 34487721 PMC8443449

[17] Wu Z Xia F Wang W Zhang K Fan M Lin R. Worldwide burden of liver cancer across childhood and adolescence, 2000-2021: a systematic analysis of the Global Burden of Disease Study 2021. EClinicalMedicine. (2024) 75:102765. doi: 10.1016/j.eclinm.2024.102765, PMID: 39170941 PMC11338123

[18] Bai Z Wang H Shen C An J Yang Z Mo X. The global, regional, and national patterns of change in the burden of nonmalignant upper gastrointestinal diseases from 1990 to 2019 and the forecast for the next decade. Int J Surg (London England). (2025) 111:80–92. doi: 10.1097/JS9.0000000000001902, PMID: 38959095 PMC11745775

[19] Wang S Dong Z Wan X. Global, regional, and national burden of inflammatory bowel disease and its associated anemia, 1990 to 2019 and predictions to 2050: An analysis of the global burden of disease study 2019. Autoimmun Rev. (2024) 23:103498. doi: 10.1016/j.autrev.2023.103498, PMID: 38052263

[20] Das Gupta P. A general method of decomposing a difference between two rates into several components. Demography. (1978) 15:99–112. doi: 10.2307/2060493, PMID: 631402

[21] World Health Organization. Handbook on health inequality monitoring with a special focus on lowand middle-income countries(2013). Available online at: https://www.who.int/publications/i/item/9789241548632 (Accessed April 15, 2025).

[22] GBD 2019 Cancer Risk Factors Collaborators. The global burden of cancer attributable to risk factors, 2010-19: a systematic analysis for the Global Burden of Disease Study 2019. Lancet (London England). (2022) 400:563–91. doi: 10.1016/S0140-6736(22)01438-6, PMID: 35988567 PMC9395583

[23] Kocarnik JM Compton K Dean FE Fu W Gaw BL Harvey JD. Cancer incidence, mortality, years of life lost, years lived with disability, and disability-Adjusted life years for 29 cancer groups from 2010 to 2019: A systematic analysis for the global burden of disease study 2019. JAMA Oncol. (2022) 8:420–44. doi: 10.1001/jamaoncol.2021.6987, PMID: 34967848 PMC8719276

[24] Field JK Vulkan D Davies MPA Baldwin DR Brain KE Devaraj A. Lung cancer mortality reduction by LDCT screening: UKLS randomised trial results and international meta-analysis. Lancet Regional Health Europe. (2021) 10:100179. doi: 10.1016/j.lanepe.2021.100179, PMID: 34806061 PMC8589726

[25] Yang JJ Yu D Wen W Shu XO Saito E Rahman S. Tobacco smoking and mortality in Asia: A pooled meta-analysis. JAMA Netw Open. (2019) 2:e191474. doi: 10.1001/jamanetworkopen.2019.1474, PMID: 30924901 PMC6450311

[26] Sun K Zhang B Lei S Zheng R Liang X Li L. Incidence, mortality, and disability-adjusted life years of female breast cancer in China, 2022. Chin Med J. (2024) 137:2429–36. doi: 10.1097/CM9.0000000000003278, PMID: 39238088 PMC11479498

[27] Zhao J Xu L Sun J Song M Wang L Yuan S. Global trends in incidence, death, burden and risk factors of early-onset cancer from 1990 to 2019. BMJ Oncol. (2023) 2:e000049. doi: 10.1136/bmjonc-2023-000049, PMID: 39886513 PMC11235000

[28] Yang X Man J Chen H Zhang T Yin X He Q. Temporal trends of the lung cancer mortality attributable to smoking from 1990 to 2017: A global, regional and national analysis. Lung Cancer (Amsterdam Netherlands). (2021) 152:49–57. doi: 10.1016/j.lungcan.2020.12.007, PMID: 33348250

[29] Kong Q Xu X Li M Meng X Zhao C Yang X. Global, regional, and national burden of myocarditis in 204 countries and territories from 1990 to 2019: updated systematic analysis. JMIR Public Health Surveil. (2024) 10:e46635. doi: 10.2196/46635, PMID: 38206659 PMC10811576

[30] Li C Lei S Ding L Xu Y Wu X Wang H. Global burden and trends of lung cancer incidence and mortality. Chin Med J. (2023) 136:1583–90. doi: 10.1097/CM9.0000000000002529, PMID: 37027426 PMC10325747

[31] Zhang C Yu L Pan X Lu Y Pan K. Disease burden comparison and associated risk factors of early- and late-onset neonatal sepsis in China and the USA, 1990-2019. Global Health Action. (2024) 17:2396734. doi: 10.1080/16549716.2024.2396734, PMID: 39229931 PMC11376289

[32] Holford TR Levy DT McKay LA Clarke L Racine B Meza R. Patterns of birth cohort-specific smoking histories, 1965-2009. Am J Prev Med. (2014) 46:e31–37. doi: 10.1016/j.amepre.2013.10.022, PMID: 24439359 PMC3951759

[33] He F Wang S Zheng R Gu J Zeng H Sun K. Trends of gastric cancer burdens attributable to risk factors in China from 2000 to 2050. Lancet Regional Health Western Pacif. (2024) 44:101003. doi: 10.1016/j.lanwpc.2023.101003, PMID: 38269331 PMC10806286

[34] Filho AM Laversanne M Ferlay J Colombet M Piñeros M Znaor A. The GLOBOCAN 2022 cancer estimates: Data sources, methods, and a snapshot of the cancer burden worldwide. Int J Cancer. (2025) 156:1336–46. doi: 10.1002/ijc.35278, PMID: 39688499

[35] Li L Shan T Zhang D Ma F. Nowcasting and forecasting global aging and cancer burden: analysis of data from the GLOBOCAN and Global Burden of Disease Study. J Natl Cancer Center. (2024) 4:223–32. doi: 10.1016/j.jncc.2024.05.002, PMID: 39281725 PMC11401500

[36] World Health Organization. Tobacco. (2021). Available online at: https://www.who.int/news-room/fact-sheets/detail/tobacco (Accessed April 18, 2025).

[37] Chen S Dai S Hou Y. Learn from tobacco to reduce betel nut use. Sci (New York NY). (2023) 382:777–8. doi: 10.1126/science.adk7903, PMID: 37972186

[38] Huang L He J. Trend analysis of hematological tumors in adolescents and young adults from 1990 to 2019 and predictive trends from 2020 to 2044: A Global Burden of Disease study. Cancer Med. (2024) 13:e70224. doi: 10.1002/cam4.70224, PMID: 39359159 PMC11447274

[39] Bray F Ferlay J Soerjomataram I Siegel RL Torre LA Jemal A. Global cancer statistics 2018: GLOBOCAN estimates of incidence and mortality worldwide for 36 cancers in 185 countries. CA: Cancer J Clin. (2018) 68:394–424. doi: 10.3322/caac.21492, PMID: 30207593

[40] World Health Organization. WHO report on the global tobacco epidemic 2019. Available online at: https://www.who.int/publications/i/item/9789241516204 (Accessed April 20, 2025).

[41] Kyrgiou M Bowden S Denny L Fagotti A Abu-Rustum NR Ramirez PT. Innovation in gynaecological cancer: highlighting global disparities. Lancet Oncol. (2024) 25:425–30. doi: 10.1016/S1470-2045(24)00137-2, PMID: 38461833

