# Supplementary material for: Global burden and cross-national inequalities of tobacco-attributable cancers in adults aged 40 and above, 1990–2021: a population-based study
Source: Front Oncol. 2025 Jul 25;15:1631356. doi: 10.3389/fonc.2025.1631356 (PMC12331488; doi:10.3389/fonc.2025.1631356)
Supplement: Supplementary file 1 [file DataSheet1.docx]

Supplementary Material

# Supplementary Data

Supplementary Material should be uploaded separately on submission. Please include any supplementary data, figures and/or tables.

Supplementary material is not typeset so please ensure that all information is clearly presented, the appropriate caption is included in the file and not in the manuscript, and that the style conforms to the rest of the article.

# Supplementary Figures and Tables

## Supplementary Figures


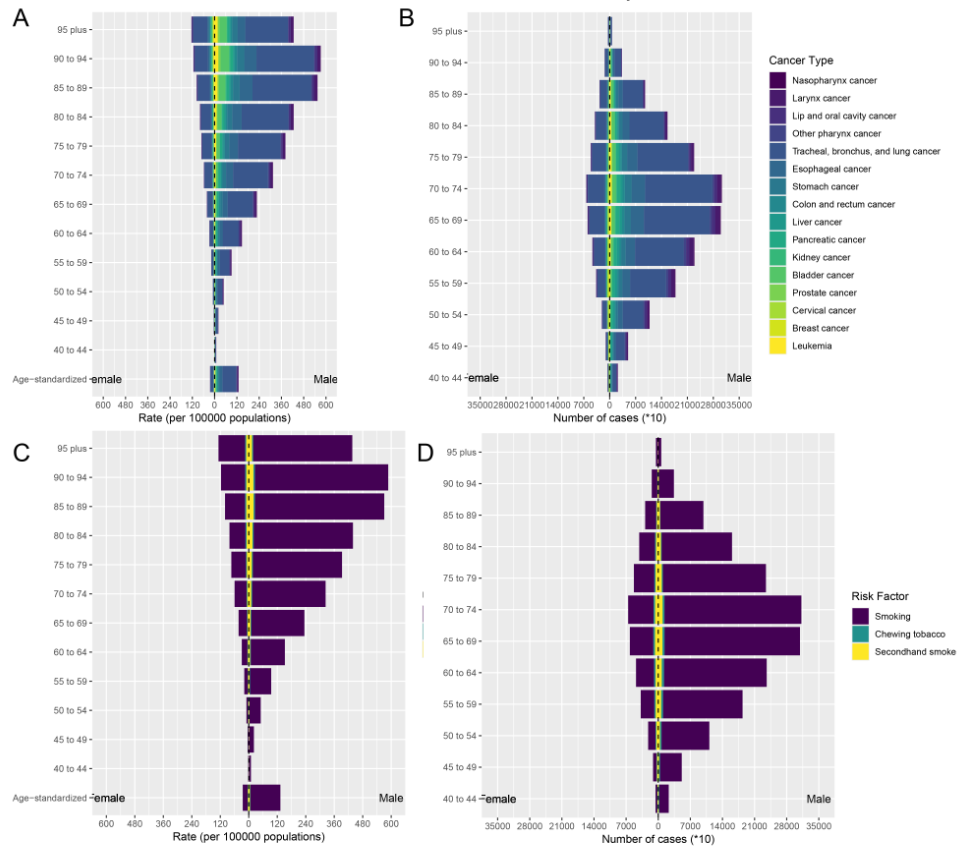


**Figure S1** The burden of tobacco-attributable cancer and cancer stratified by age and sex. (A) ASR-deaths for cancers attributable to tobacco. (B) Number of death cases for cancers attributable to tobacco. (C) ASR-deaths for tobacco-attributable cancers. (D) Number of deaths cases for tobacco-attributable cancers.


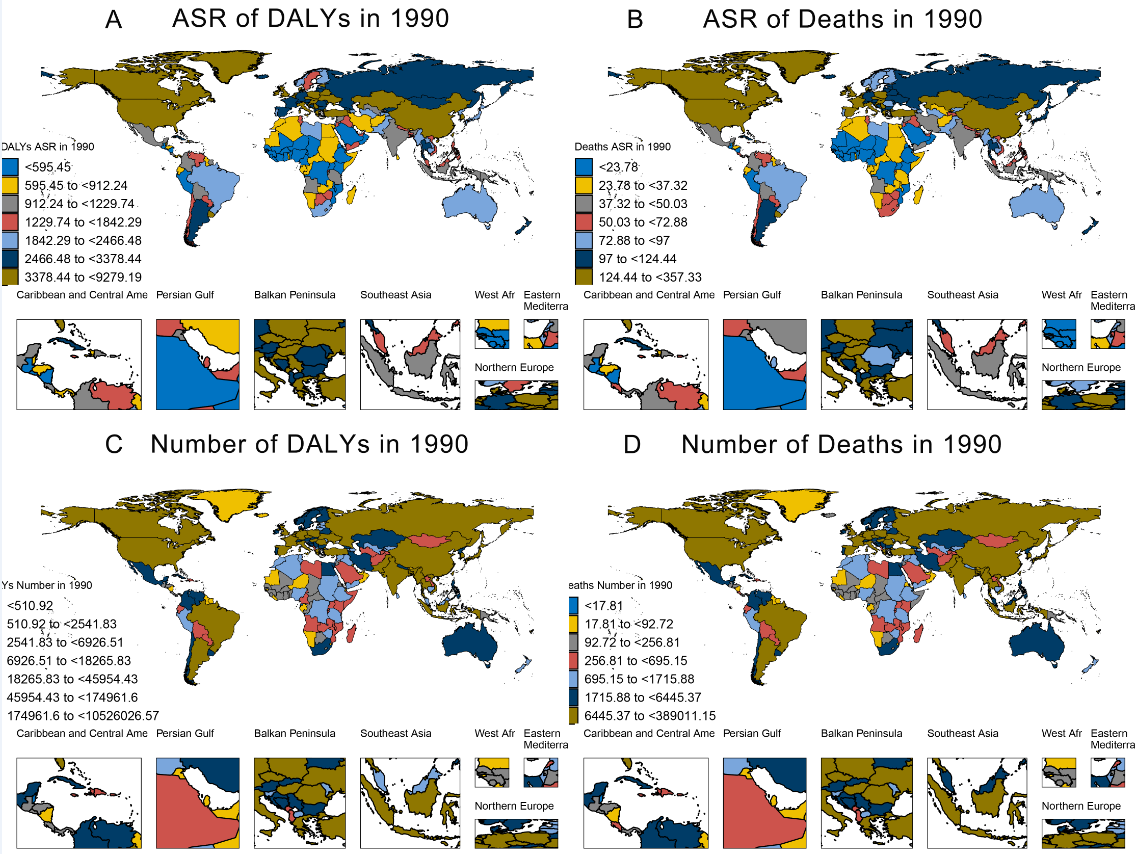


**Figure S2** The heatmap for the global burden of cancer attributable to tobacco in 1990. (A) ASR of DALYs.(B) ASR of Deaths.(C)Number of DALYs.(D)Number of Deaths.


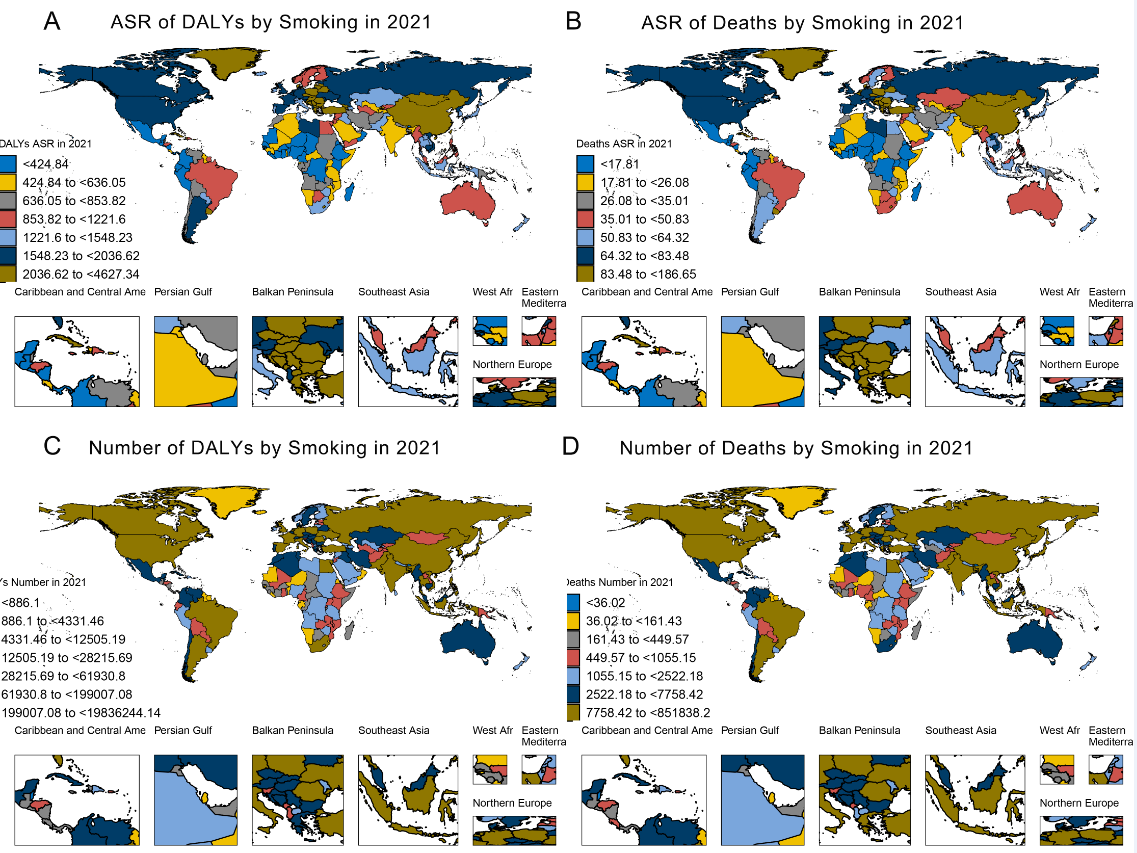


**Figure S3** The heatmap for the global burden of cancers attributable to smoking in 2021. (A) ASR of DALYs. (B) ASR of Deaths. (C)Number of DALYs. (D)Number of Deaths.
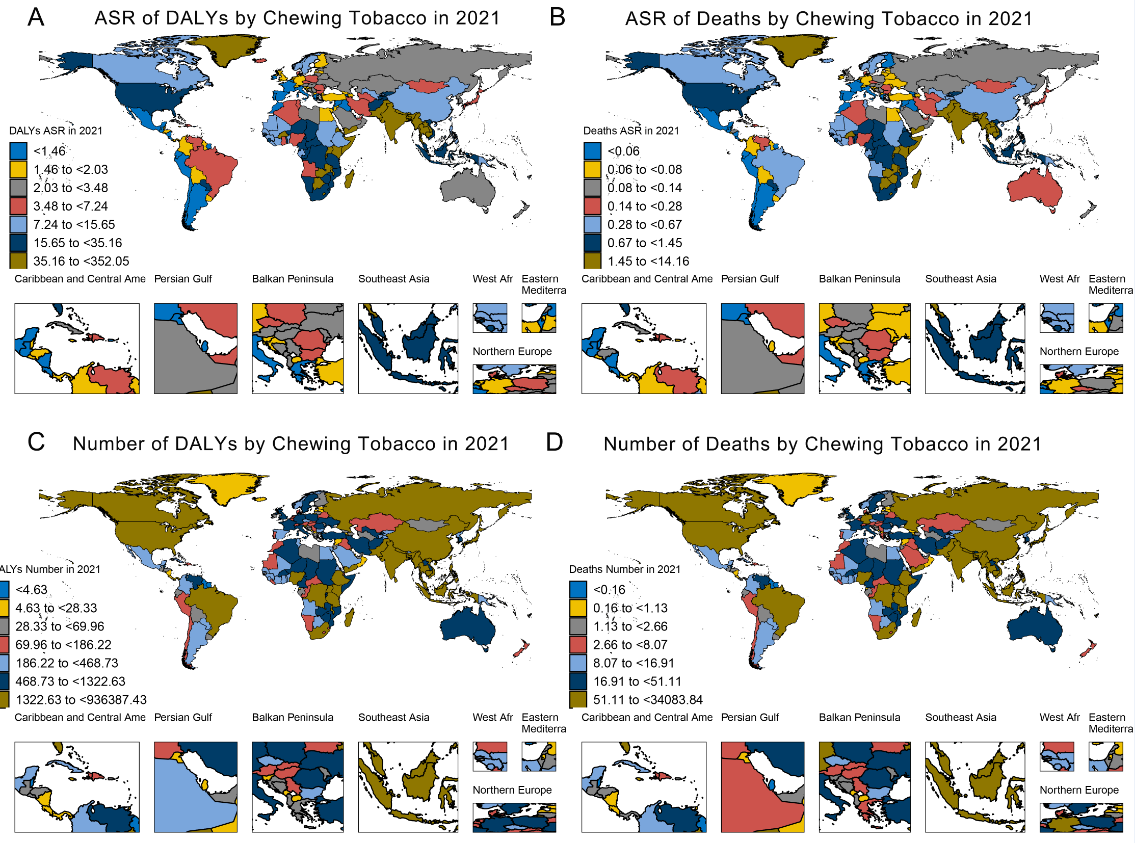


**Figure S4** The heatmap for the global burden of cancers attributable to chewing tobacco in 2021. (A) ASR of DALYs. (B) ASR of Deaths.(C)Number of DALYs.(D)Number of Deaths.


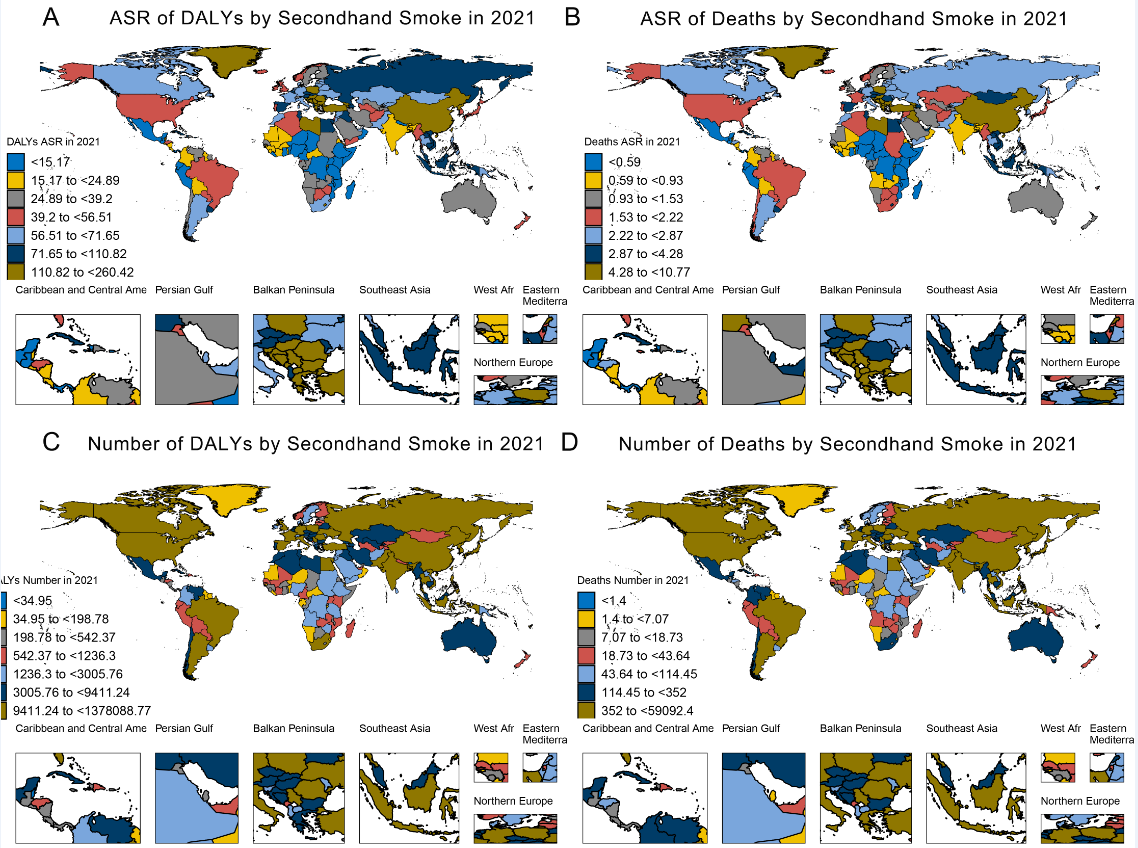


**Figure S5** The heatmap for the global burden of cancers attributable to secondhand smoke in 2021. (A) ASR of DALYs. (B) ASR of Deaths.(C)Number of DALYs.(D)Number of Deaths.
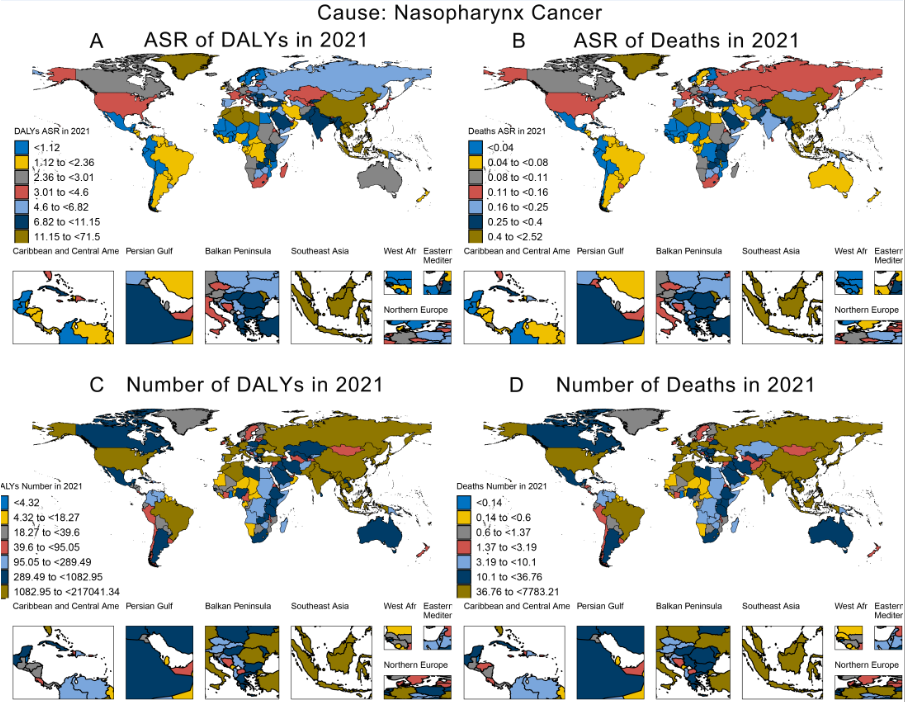


**Figure S6** The heatmap for the global burden of nasopharynx cancer in 2021. (A) ASR of DALYs.(B) ASR of Deaths.(C)Number of DALYs.(D)Number of Deaths.


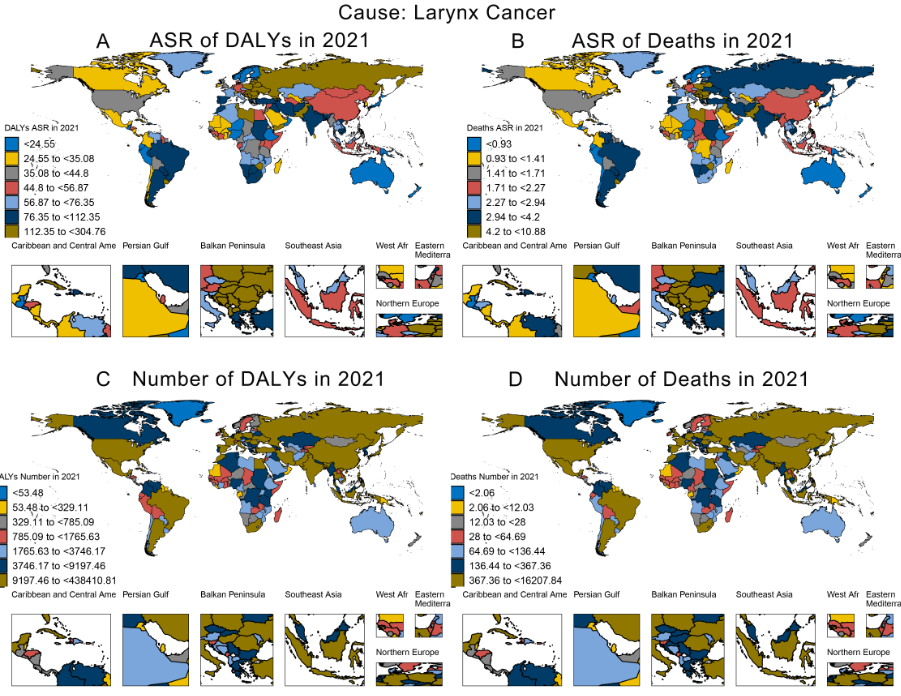


**Figure S7** The heatmap for the global burden of larynx cancer in 2021. (A) ASR of DALYs. (B) ASR of Deaths.(C)Number of DALYs.(D)Number of Deaths.


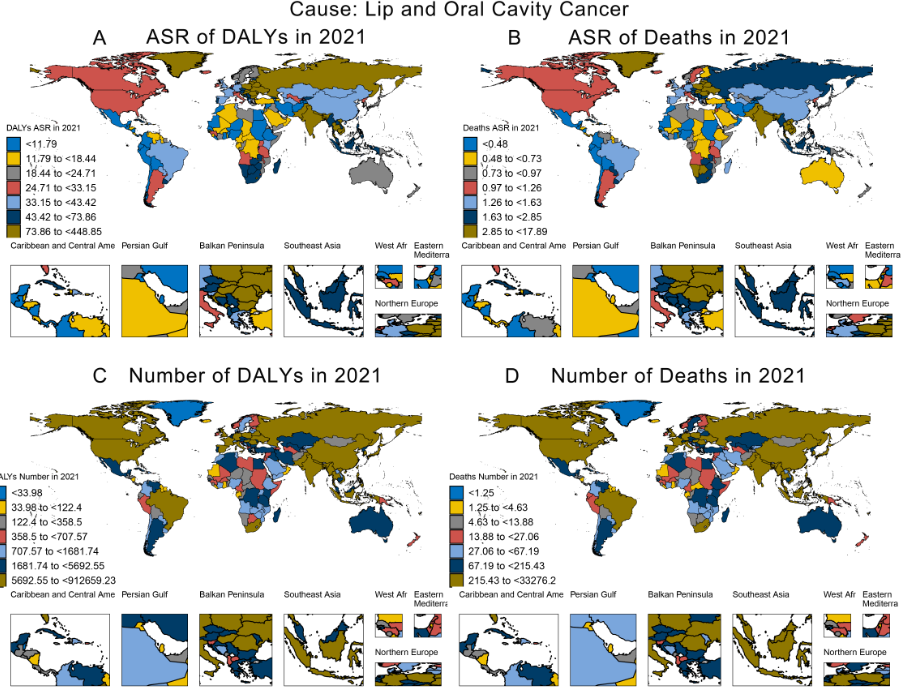


**Figure S8** The heatmap for the global burden of lip and oral cavity cancer in 2021. (A) ASR of DALYs. (B) ASR of Deaths. (C)Number of DALYs.(D)Number of Deaths.


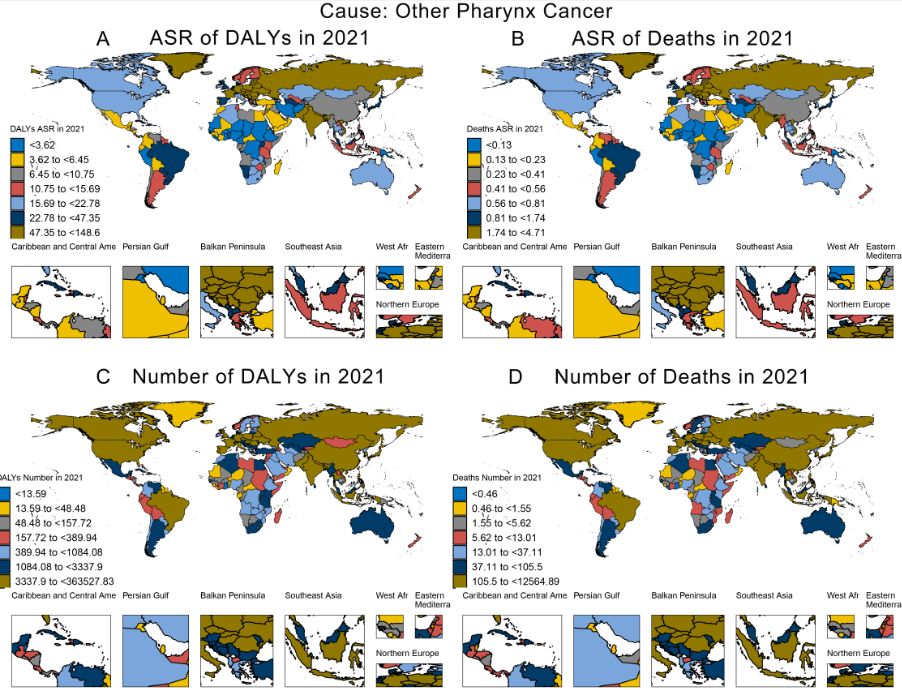


**Figure S9** The heatmap for the global burden of other pharynx cancer in 2021. (A) ASR of DALYs. (B) ASR of Deaths. (C)Number of DALYs. (D)Number of Deaths.


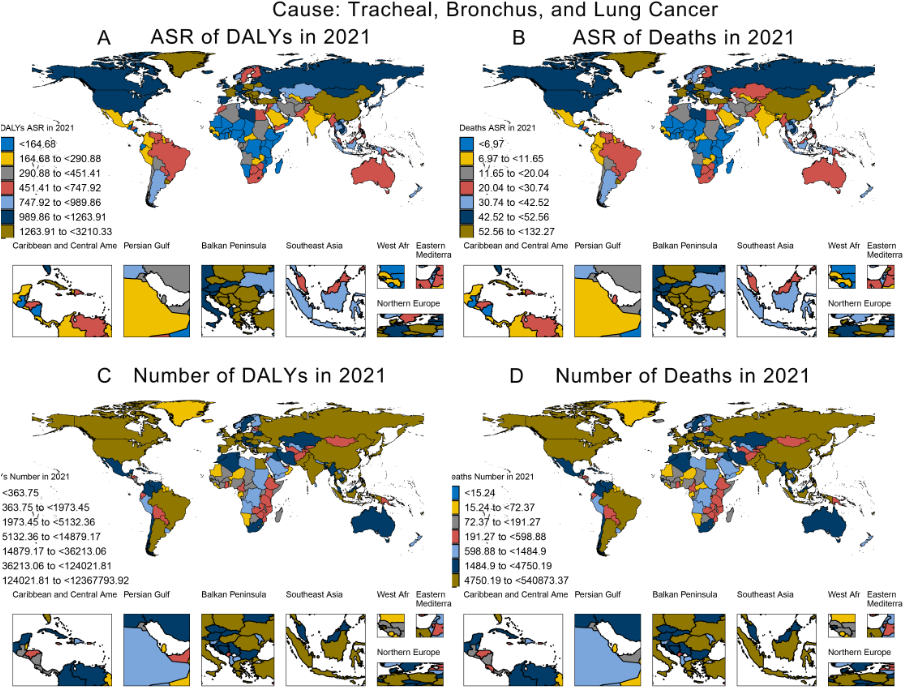


**Figure S10** The heatmap for the global burden of tracheal, bronchus and lung cancer in 2021. (A) ASR of DALYs. (B) ASR of Deaths. (C)Number of DALYs. (D)Number of Deaths.


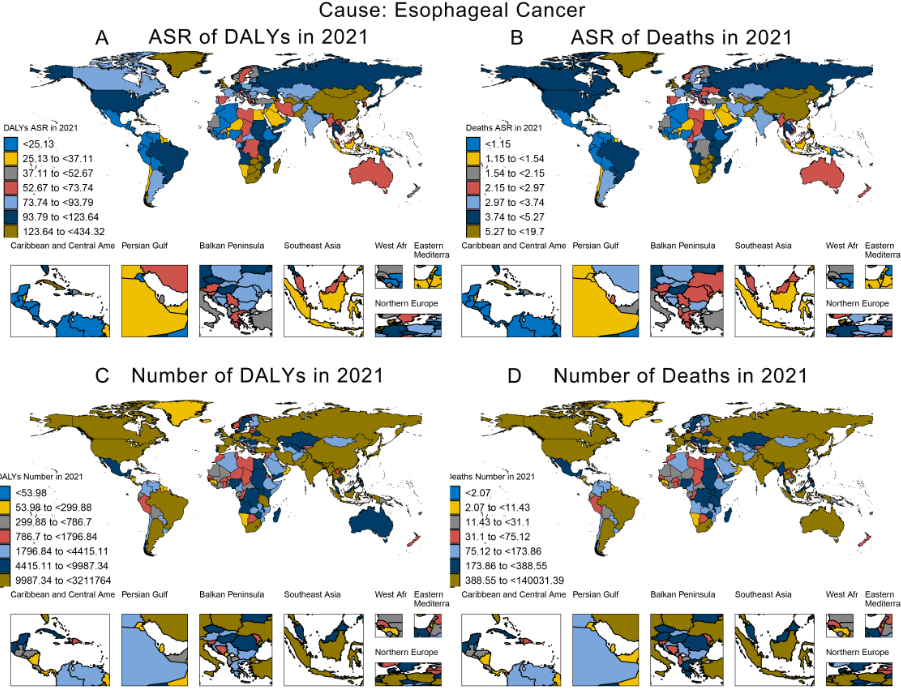


**Figure S11** The heatmap for the global burden of esophageal cancer in 2021. (A) ASR of DALYs. (B) ASR of Deaths. (C)Number of DALYs. (D)Number of Deaths.


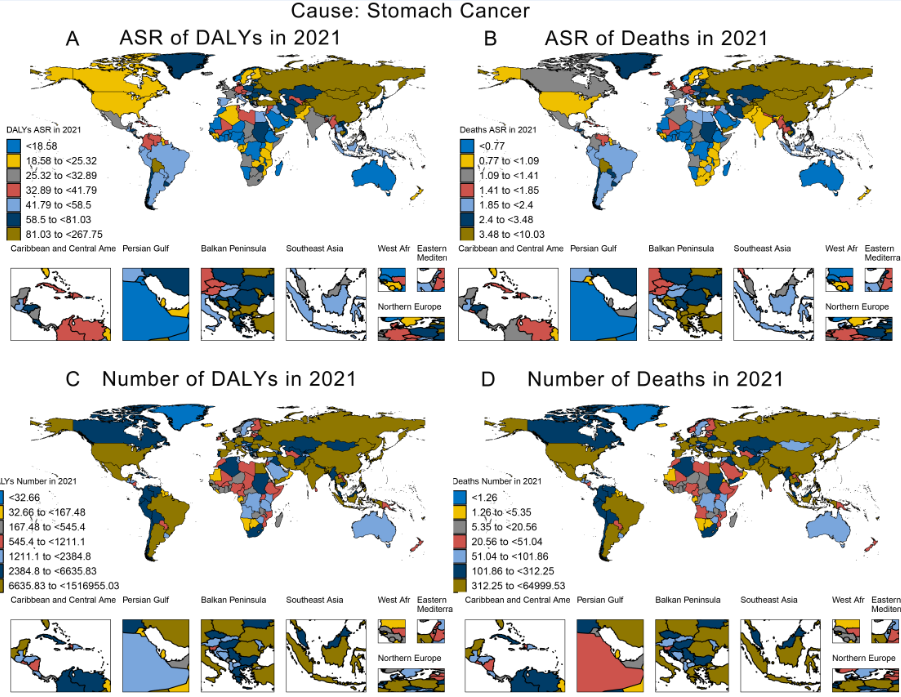


**Figure S12** The heatmap for the global burden of stomach cancer in 2021. (A) ASR of DALYs. (B) ASR of Deaths. (C)Number of DALYs. (D)Number of Deaths.


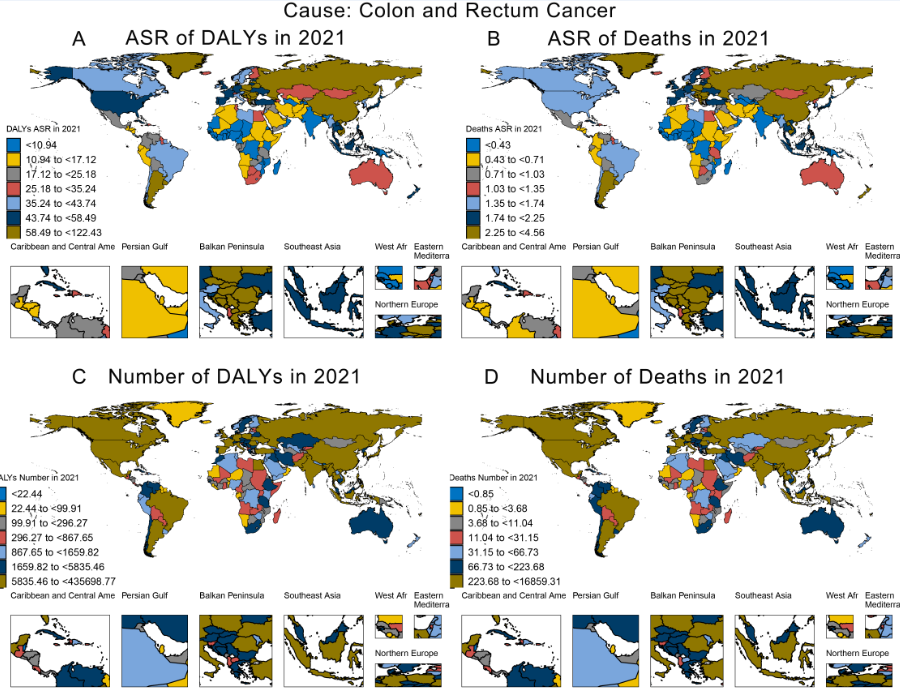


**Figure S13** The heatmap for the global burden of colon and rectum cancer in 2021. (A) ASR of DALYs. (B) ASR of Deaths. (C)Number of DALYs. (D)Number of Deaths.


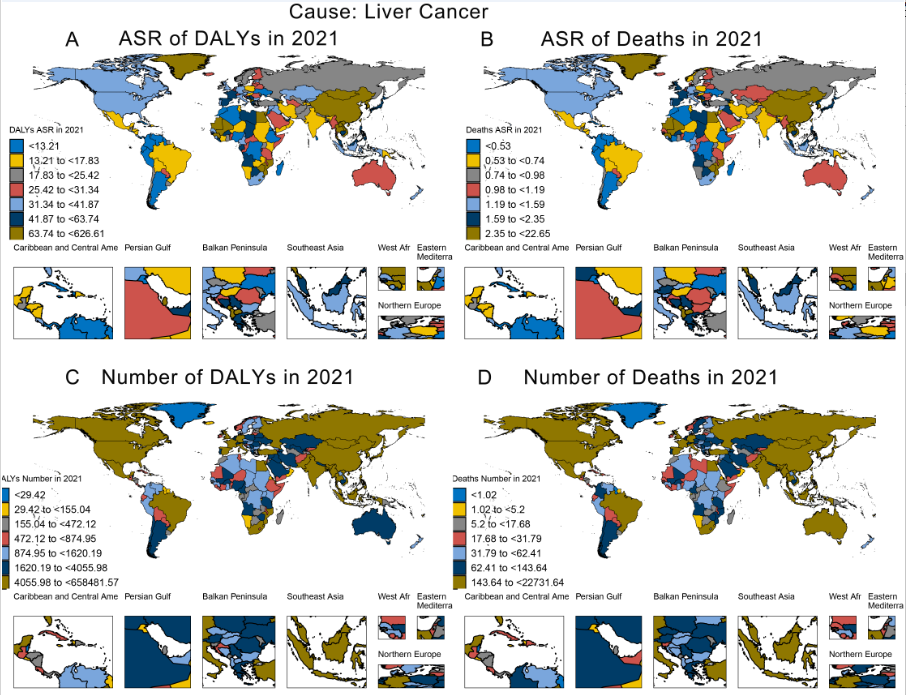


**Figure S14** The heatmap for the global burden of liver cancer in 2021. (A) ASR of DALYs. (B) ASR of Deaths. (C)Number of DALYs. (D)Number of Deaths.


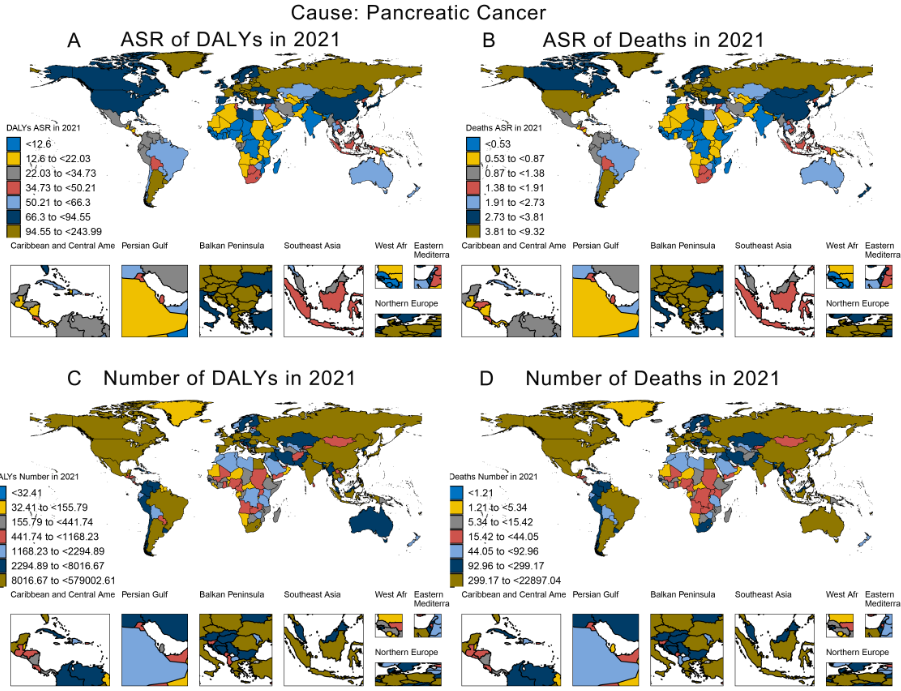


**Figure S15** The heatmap for the global burden of pancreatic cancer in 2021. (A) ASR of DALYs. (B) ASR of Deaths. (C)Number of DALYs. (D)Number of Deaths.


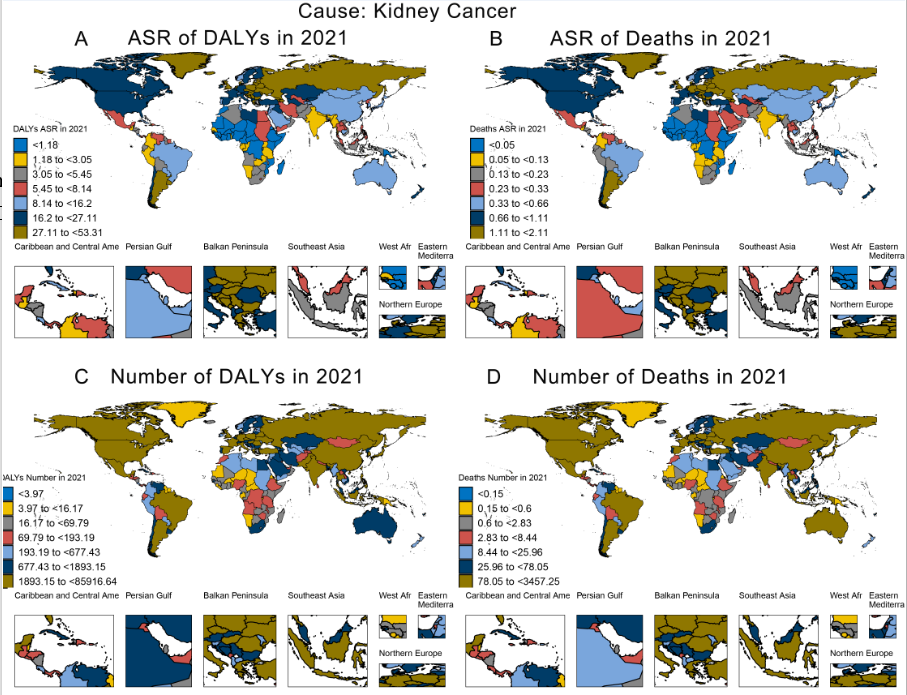


**Figure S16** The heatmap for the global burden of kidney cancer in 2021. (A) ASR of DALYs. (B) ASR of Deaths. (C)Number of DALYs. (D)Number of Deaths.


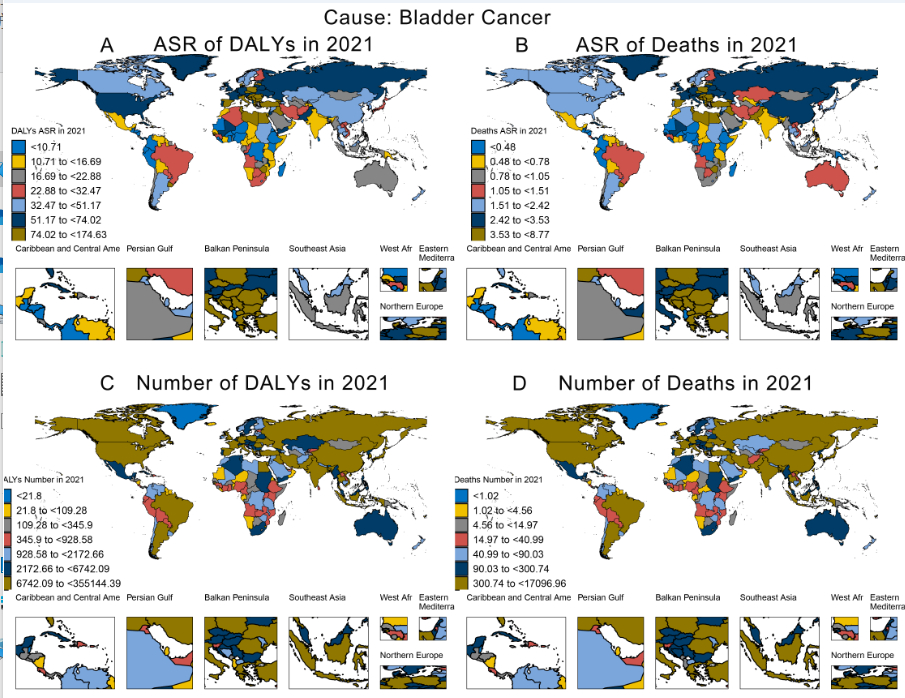


**Figure S17** The heatmap for the global burden of bladder cancer in 2021. (A) ASR of DALYs. (B) ASR of Deaths. (C)Number of DALYs. (D)Number of Deaths.


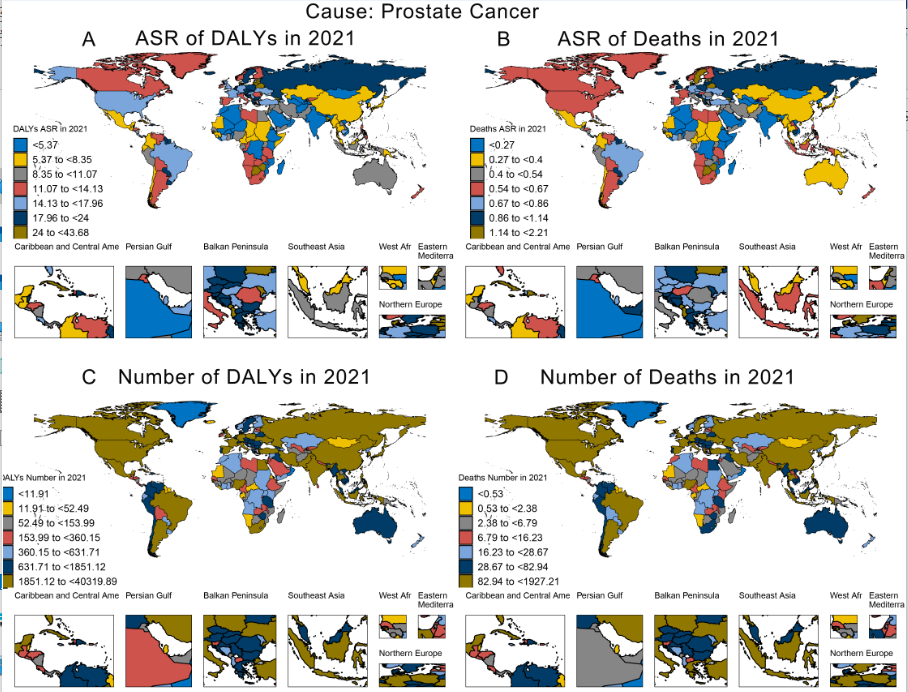


**Figure S18** The heatmap for the global burden of prostate cancer in 2021. (A) ASR of DALYs. (B) ASR of Deaths. (C)Number of DALYs. (D)Number of Deaths.


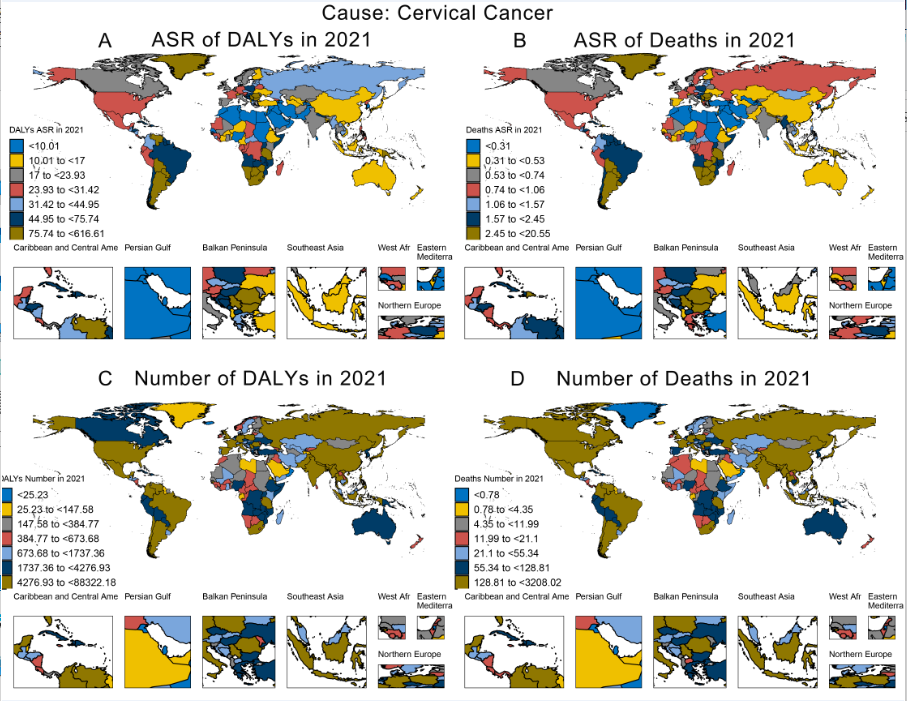


**Figure S19** The heatmap for the global burden of cervical cancer in 2021. (A) ASR of DALYs. (B) ASR of Deaths. (C)Number of DALYs. (D)Number of Deaths.


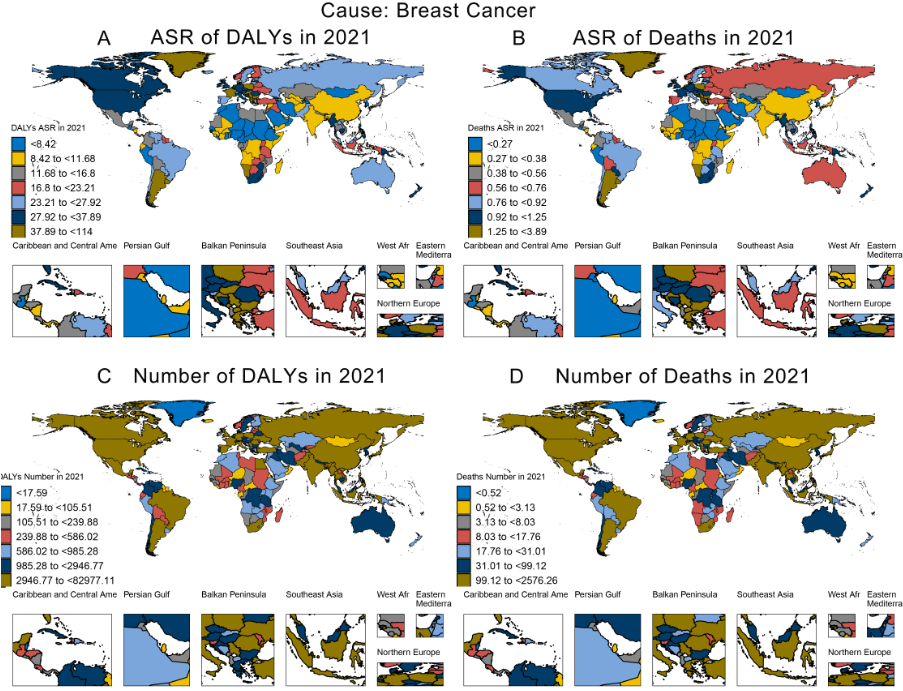


**Figure S20** The heatmap for the global burden of breast cancer in 2021. (A) ASR of DALYs. (B) ASR of Deaths. (C)Number of DALYs. (D)Number of Deaths.


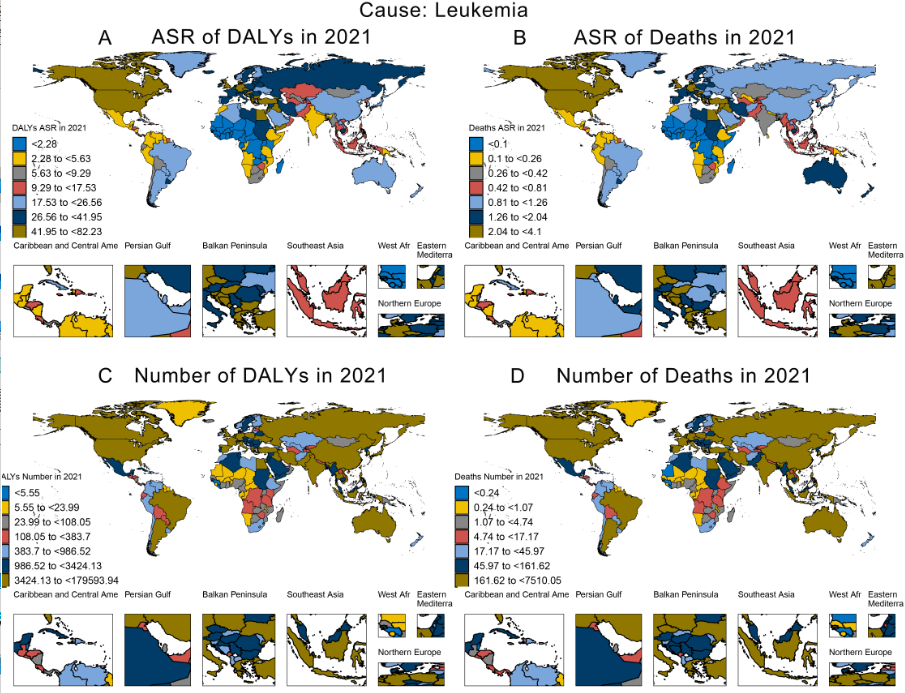


**Figure S21** The heatmap for the global burden of leukemia in 2021. (A) ASR of DALYs. (B) ASR of Deaths. (C)Number of DALYs. (D)Number of Deaths.

**2.2 Supplementary Figures**

**Table S1** Number and ASR of deaths for tobacco-attributable cancer in individuals aged 40 years and older in 2021, with trends from 1990 to 2021 and projections for 2030.

|  | 2021 | | 1990-2021 |
| --- | --- | --- | --- |
|  | Deaths cases | ASR-Deaths per 100,000 (95% UI) | EAPC of Deaths |
| Global | 2092020.63 (1719655.29 to 2487163.1) | 72.36 (59.4 to 86.09) | -1.18 (-1.23 to -1.13) |
| Global Prediction of 2030 | 2418390.67 (2023946.62 to 2812834.72) | 64.59 (54.04 to 75.15) |  |
| Sex |  |  |  |
| Male | 1709418.87 (1420633.58 to 2032066.7) | 129.22 (107.15 to 153.76) | -1.26 (-1.3 to -1.22) |
| Male Prediction of 2030 | 1990528.50 (1657747.43 to 2323309.58) | 115.75 (97.16 to 134.35) |  |
| Female | 382601.76 (283618.41 to 491560.47) | 24.59 (18.22 to 31.6) | -1.04 (-1.13 to -0.94) |
| Female Prediction of 2030 | 427862.17 (366199.19 to 489525.14) | 21.02 (18.13 to 23.91) | -1.04 (-1.13 to -0.94) |
| Age (years) |  |  |  |
| 40-44 years | 28050.38 (21737.64 to 34448.86) | 5.61 (4.35 to 6.89) | -2.8  (-2.93 to -2.66) |
| 45-49 years | 60724.69 (47958.14 to 73965.3) | 12.82 (10.13 to 15.62) | -2.39  (-2.56 to -2.23) |
| 50-54 years | 129111.5 (103593.29 to 156195.9) | 29.02 (23.28 to 35.11) | -2.16  (-2.29 to -2.04) |
| 55-59 years | 214467.54 (177040.08 to 255449.67) | 54.2 (44.74 to 64.55) | -1.75  (-1.83 to -1.68) |
| 60-64 years | 275764.95 (231982.81 to 322915.54) | 86.16 (72.48 to 100.9) | -1.52  (-1.59 to -1.45) |
| 65-69 years | 359819.1 (303244.93 to 421103.19) | 130.44 (109.93 to 152.66) | -1.37  (-1.43 to -1.32) |
| 70-74 years | 366558.75 (303796.16 to 432866.68) | 178.08 (147.59 to 210.29) | -1.08  (-1.17 to -0.99) |
| 75-79 years | 279466.2 (229193.31 to 332305.9) | 211.9 (173.78 to 251.97) | -0.64  (-0.75 to -0.52) |
| 80-84 years | 196317.2 (159288.31 to 235565.95) | 224.15 (181.87 to 268.96) | -0.22  (-0.34 to -0.09) |
| 85-89 years | 123271.96 (98559.63 to 148927.45) | 269.61 (215.56 to 325.73) | 0.18  (0.06 to 0.31) |
| 90-94 years | 47118.34 (35608.48 to 58675.86) | 263.39 (199.05 to 327.99) | 0.12  (0.07 to 0.18) |
| 95+ years | 11350 (7652.49 to 14742.81) | 208.25 (140.4 to 270.49) | 0.03  (-0.06 to 0.12) |
| SDI |  |  |  |
| High | 543640.85 (447861.95 to 637650.96) | 75.87 (62.88 to 88.58) | -1.8  (-1.88 to -1.72) |
| High-middle | 683108.9 (556805.01 to 828807.29) | 101.28 (82.43 to 122.96) | -0.87  (-0.94 to -0.8) |
| Middle | 640328.57 (505082.8 to 793802.16) | 72.21 (56.91 to 89.5) | -0.81  (-0.86 to -0.76) |
| Low-middle | 186878.33 (153916.89 to 220232.11) | 39.58 (32.59 to 46.66) | -0.51  (-0.54 to -0.48) |
| Low | 36049.05 (27908.57 to 44805.68) | 22.3 (17.34 to 27.63) | -0.85  (-0.92 to -0.78) |
| GBD region |  |  |  |
| High-income | 538977.53 (442625.45 to 634457.01) | 72.32 (59.88 to 84.57) | -1.88  (-1.95 to -1.8) |
| Southeast Asia, East Asia, and Oceania | 1021565.51 (794893.85 to 1287852.81) | 108.08 (84.1 to 136.02) | -0.41  (-0.52 to -0.3) |
| South Asia | 175390.57 (142360.95 to 210366.14) | 36.08 (29.3 to 43.26) | -0.87  (-0.94 to -0.8) |
| Central Europe, Eastern Europe, and Central Asia | 179452.84 (152087.5 to 205467.39) | 81.25 (68.77 to 93.11) | -1.35  (-1.46 to -1.25) |
| North Africa and Middle East | 75476.16 (59492.43 to 92517.97) | 52.15 (40.96 to 64.13) | -0.7  (-0.79 to -0.6) |
| Latin America and Caribbean | 71813.39 (57497.05 to 86580.15) | 34.73 (27.76 to 41.91) | -2.12  (-2.2 to -2.04) |
| Sub-Saharan Africa | 29344.63 (22023.94 to 36964.11) | 18.81 (14.16 to 23.69) | -1.01  (-1.14 to -0.88) |
| Carcinogen risk |  |  |  |
| Smoking | 1998048.77 (1661002.11 to 2360756.63) | 69.1 (57.36 to 81.7) | -1.22  (-1.27 to -1.17) |
| Chewing tobacco | 55065.2 (41953.39 to 69577.14) | 1.9 (1.45 to 2.4) | -0.03  (-0.1 to 0.05) |
| Secondhand smoke | 103604.99 (10278.91 to 200698.23) | 3.59 (0.36 to 6.96) | -0.89  (-0.95 to -0.83) |
| Cancer |  |  |  |
| Nasopharynx cancer | 13039.35 (9390.93 to 17135.31) | 0.44 (0.32 to 0.58) | -2.36  (-2.56 to -2.16) |
| Larynx cancer | 77403.96 (68208.1 to 86732.35) | 2.65 (2.33 to 2.97) | -2.06  (-2.13 to -1.99) |
| Lip and oral cavity cancer | 80012.84 (63469.02 to 96811.12) | 2.75 (2.18 to 3.33) | -0.51  (-0.55 to -0.46) |
| Other pharynx cancer | 35676.73 (27973.99 to 43204.53) | 1.21 (0.95 to 1.47) | -0.68  (-0.75 to -0.62) |
| Tracheal, bronchus, and lung cancer | 1231763.39 (1056126.29 to 1423103.93) | 42.6 (36.48 to 49.25) | -0.94  (-1.02 to -0.87) |
| Esophageal cancer | 218166.81 (168502.21 to 273858.51) | 7.54 (5.82 to 9.47) | -1.31  (-1.42 to -1.2) |
| Stomach cancer | 106835.02 (81918.8 to 138775.62) | 3.71 (2.84 to 4.81) | -2.61  (-2.66 to -2.56) |
| Colon and rectum cancer | 46766.17 (28955.94 to 65771.8) | 1.62 (1 to 2.28) | -1.25  (-1.28 to -1.22) |
| Liver cancer | 51612.84 (17314.58 to 87499.11) | 1.77 (0.59 to 3) | -0.78  (-0.89 to -0.67) |
| Pancreatic cancer | 71346.94 (60943.28 to 82930.75) | 2.46 (2.1 to 2.86) | -0.47  (-0.5 to -0.45) |
| Kidney cancer | 16162.93 (9582.61 to 23385.15) | 0.56 (0.33 to 0.82) | -0.93  (-1.03 to -0.82) |
| Bladder cancer | 58588.04 (48306.98 to 71116.26) | 2.08 (1.71 to 2.53) | -1.71  (-1.77 to -1.65) |
| Prostate cancer | 12971.44 (5821.44 to 21817.31) | 0.46 (0.21 to 0.78) | -2.19  (-2.29 to -2.09) |
| Cervical cancer | 21419.32 (12399.11 to 32008.87) | 0.73 (0.42 to 1.09) | -2.39  (-2.42 to -2.36) |
| Breast cancer | 16472.96 (5814.99 to 27510.06) | 0.57 (0.2 to 0.95) | -1.82  (-1.88 to -1.76) |
| Leukemia | 33781.89 (11776.44 to 58434.2) | 1.2 (0.41 to 2.08) | -1.5  (-1.58 to -1.42) |
|  |  |  |  |

**Table S2** The decomposition of aging, population, and epidemiological change for tobacco-attributable cancers from 1990 to 2021

| Measure | Location | Overall difference | Aging | Population | Epidemiological change | percent change of Aging | percent change of Population | percent change of Epidemiological change |
| --- | --- | --- | --- | --- | --- | --- | --- | --- |
| DALYs | Global | 13967495.41 | 2072475.22 | 31936601.69 | -20041581.50 | 14.84 | 228.65 | -143.49 |
|  | High SDI | -291229.99 | 1030259.98 | 6111069.01 | -7432558.97 | -353.76 | -2098.37 | 2552.13 |
|  | High-middle SDI | 4321432.03 | 593723.07 | 9080089.97 | -5352381.02 | 13.74 | 210.12 | -123.86 |
|  | Middle SDI | 7114668.91 | 974208.03 | 10619659.86 | -4479198.98 | 13.69 | 149.26 | -62.96 |
|  | Low-middle SDI | 2411060.53 | 98088.64 | 3056681.04 | -743709.15 | 4.07 | 126.78 | -30.85 |
|  | Low SDI | 407127.60 | -35277.89 | 685600.31 | -243194.82 | -8.67 | 168.40 | -59.73 |
|  | High-income | -1159824.64 | 1034024.78 | 5601582.09 | -7795431.51 | -89.15 | -482.97 | 672.12 |
|  | Southeast Asia, East Asia, and Oceania | 11966915.39 | 1873812.27 | 14979352.61 | -4886249.49 | 15.66 | 125.17 | -40.83 |
|  | South Asia | 2255722.93 | 172039.52 | 3098683.40 | -1014999.99 | 7.63 | 137.37 | -45.00 |
|  | Central Europe, Eastern Europe, and Central Asia | -992663.20 | -9942.09 | 1337510.34 | -2320231.44 | 1.00 | -134.74 | 233.74 |
|  | North Africa and Middle East | 979983.49 | -58117.97 | 1552482.96 | -514381.50 | -5.93 | 158.42 | -52.49 |
|  | Latin America and Caribbean | 558216.53 | 118539.89 | 1511200.06 | -1071523.43 | 21.24 | 270.72 | -191.95 |
|  | Sub-Saharan Africa | 359144.91 | -38025.71 | 596093.23 | -198922.61 | -10.59 | 165.98 | -55.39 |
| Deaths | Global | 741754.41 | 142577.54 | 1265756.57 | -666579.70 | 19.22 | 170.64 | -89.87 |
|  | High SDI | 56888.84 | 70626.36 | 263005.36 | -276742.88 | 124.15 | 462.31 | -486.46 |
|  | High-middle SDI | 242299.55 | 44433.51 | 352194.73 | -154328.69 | 18.34 | 145.36 | -63.69 |
|  | Middle SDI | 331557.20 | 60495.18 | 409155.07 | -138093.05 | 18.25 | 123.40 | -41.65 |
|  | Low-middle SDI | 95173.23 | 7553.90 | 111455.18 | -23835.85 | 7.94 | 117.11 | -25.04 |
|  | Low SDI | 15484.65 | -1200.64 | 24355.58 | -7670.29 | -7.75 | 157.29 | -49.53 |
|  | High-income | 20241.95 | 75776.44 | 242824.42 | -298358.92 | 374.35 | 1199.61 | -1473.96 |
|  | Southeast Asia, East Asia, and Oceania | 569887.79 | 116697.18 | 589251.37 | -136060.76 | 20.48 | 103.40 | -23.88 |
|  | South Asia | 89912.90 | 12179.04 | 111326.81 | -33592.95 | 13.55 | 123.82 | -37.36 |
|  | Central Europe, Eastern Europe, and Central Asia | -16728.46 | 5823.42 | 48407.01 | -70958.89 | -34.81 | -289.37 | 424.18 |
|  | North Africa and Middle East | 39654.43 | -2019.99 | 57715.15 | -16040.73 | -5.09 | 145.55 | -40.45 |
|  | Latin America and Caribbean | 26180.87 | 7334.34 | 59240.06 | -40393.54 | 28.01 | 226.27 | -154.29 |
|  | Sub-Saharan Africa | 12604.94 | -1841.48 | 20983.09 | -6536.67 | -14.61 | 166.47 | -51.86 |

**Table S3** The slope indicies of ASR for tobacco-attributable cancers from 1990 to 2021

| Location | Year | Measure | Slope Indicies | | |
| --- | --- | --- | --- | --- | --- |
|  |  |  | lm (95%CI) | ncvTest p-value | rlm (95%CI) |
| Global | 1990 | DALYs | 2782.79(2356.3 to 3209.27) | <0.001 | 2653.85(2263.94 to 3043.76) |
|  |  | Deaths | 108.38(92.4 to 124.36) | <0.001 | 104.48(89.78 to 119.18) |
|  | 2021 | DALYs | 1255.95(965.92 to 1545.99) | <0.001 | 1178.13(930.11 to 1426.15) |
|  |  | Deaths | 52.27(41.07 to 63.47) | <0.001 | 49.7(39.64 to 59.75) |
| Low SDI | 1990 | DALYs | 618.4(221.9 to 1014.91) | 0.0568 | 546.65(187.01 to 906.29) |
|  |  | Deaths | 22.73(7.36 to 38.11) | 0.0526 | 19.72(6.36 to 33.09) |
|  | 2021 | DALYs | 509.23(212.96 to 805.51) | 0.0222 | 458.38(200.63 to 716.13) |
|  |  | Deaths | 20.71(8.93 to 32.48) | 0.0197 | 18.87(7.29 to 30.45) |
| Low-middle SDI | 1990 | DALYs | 256.21(-298.59 to 811) | 0.9323 | 229.3(-352.02 to 810.62) |
|  |  | Deaths | 7.89(-13.11 to 28.88) | 0.7031 | 9.06(-13.53 to 31.64) |
|  | 2021 | DALYs | 231.9(-281.83 to 745.63) | 0.8852 | 210.22(-294.82 to 715.26) |
|  |  | Deaths | 7.76(-12.09 to 27.62) | 0.6676 | 7.42(-13.55 to 28.4) |
| Middle SDI | 1990 | DALYs | 691.98(117.6 to 1266.36) | 0.0125 | 615.67(21.87 to 1209.47) |
|  |  | Deaths | 24.26(1.93 to 46.58) | 0.0457 | 21.43(-2.96 to 45.82) |
|  | 2021 | DALYs | -126.73(-619.62 to 366.16) | 0.7190 | -133.91(-592.33 to 324.5) |
|  |  | Deaths | -4.73(-24.62 to 15.16) | 0.5230 | -6.09(-24.52 to 12.33) |
| High-middle SDI | 1990 | DALYs | 1636.86(-173.39 to 3447.12) | 0.8175 | 1678.49(-320.1 to 3677.08) |
|  |  | Deaths | 60.15(-5.35 to 125.65) | 0.5951 | 68.01(-4.93 to 140.96) |
|  | 2021 | DALYs | -261.85(-1462.41 to 938.72) | 0.6149 | -392.52(-1840.55 to 1055.51) |
|  |  | Deaths | -14.09(-59.3 to 31.11) | 0.8178 | -20.25(-76.24 to 35.75) |
| High SDI | 1990 | DALYs | 654.76(-699.65 to 2009.17) | 0.1342 | 757.05(-344.05 to 1858.14) |
|  |  | Deaths | 33.28(-17.73 to 84.28) | 0.1989 | 35.65(-3.14 to 74.45) |
|  | 2021 | DALYs | 167.66(-563.04 to 898.36) | 0.1272 | 216.19(-345.33 to 777.7) |
|  |  | Deaths | 13.83(-14.87 to 42.53) | 0.1341 | 16.4(-5.22 to 38.03) |

**Table S4** The intercept indicies of ASR for tobacco-attributable cancers from 1990 to 2021

| Location | Year | Measure | Intercept Indicies | | |
| --- | --- | --- | --- | --- | --- |
|  |  |  | lm (95%CI) | ncvTest p-value | rlm (95%CI) |
| Global | 1990 | DALYs | 562.33(326.78 to 797.89) | <0.001 | 568.22(352.86 to 783.58) |
|  |  | Deaths | 21.95(13.12 to 30.77) | <0.001 | 21.93(13.82 to 30.05) |
|  | 2021 | DALYs | 691.09(526.6 to 855.57) | <0.001 | 631.62(490.96 to 772.27) |
|  |  | Deaths | 27.38(21.03 to 33.73) | <0.001 | 25.41(19.7 to 31.11) |
| Low SDI | 1990 | DALYs | 377.22(136.63 to 617.81) | 0.0568 | 378.22(159.99 to 596.44) |
|  |  | Deaths | 14.96(5.63 to 24.29) | 0.0526 | 15.05(6.93 to 23.16) |
|  | 2021 | DALYs | 329.63(167.48 to 491.77) | 0.0222 | 336.43(195.37 to 477.5) |
|  |  | Deaths | 12.77(6.32 to 19.21) | 0.0197 | 13.04(6.7 to 19.38) |
| Low-middle SDI | 1990 | DALYs | 1031.71(620.81 to 1442.61) | 0.9323 | 1007.37(576.83 to 1437.91) |
|  |  | Deaths | 41.57(26.02 to 57.12) | 0.7031 | 38.77(22.04 to 55.5) |
|  | 2021 | DALYs | 918.77(635.65 to 1201.89) | 0.8852 | 876.31(597.98 to 1154.65) |
|  |  | Deaths | 37.54(26.6 to 48.49) | 0.6676 | 35.42(23.86 to 46.98) |
| Middle SDI | 1990 | DALYs | 1103.73(730.52 to 1476.94) | 0.0125 | 1121.5(735.67 to 1507.33) |
|  |  | Deaths | 46.07(31.56 to 60.57) | 0.0457 | 46.41(30.57 to 62.26) |
|  | 2021 | DALYs | 1142.34(827.95 to 1456.72) | 0.7190 | 1091.47(799.07 to 1383.86) |
|  |  | Deaths | 46.7(34.01 to 59.38) | 0.5230 | 45.09(33.34 to 56.84) |
| High-middle SDI | 1990 | DALYs | 1359.05(20.27 to 2697.83) | 0.8175 | 1314.73(-163.34 to 2792.79) |
|  |  | Deaths | 53.39(4.95 to 101.83) | 0.5951 | 47.11(-6.83 to 101.06) |
|  | 2021 | DALYs | 2030.79(1074.52 to 2987.06) | 0.6149 | 2084.22(930.83 to 3237.6) |
|  |  | Deaths | 82.97(46.96 to 118.98) | 0.8178 | 85.83(41.23 to 130.43) |
| High SDI | 1990 | DALYs | 2809.47(2166.33 to 3452.61) | 0.1342 | 2660.67(2137.81 to 3183.52) |
|  |  | Deaths | 108.04(83.81 to 132.26) | 0.1989 | 103.39(84.97 to 121.81) |
|  | 2021 | DALYs | 1593.19(1192.73 to 1993.65) | 0.1272 | 1471.8(1164.07 to 1779.54) |
|  |  | Deaths | 64.06(48.33 to 79.79) | 0.1341 | 59.09(47.24 to 70.95) |

**Table S5** The concentration indicies of ASR for tobacco-attributable cancers from 1990 to 2021

| Location | Year | Measure | Concentration Indicies | | |
| --- | --- | --- | --- | --- | --- |
|  |  |  | concentration Indicies (95%CI) | SE | p-value |
| Global | 1990 | DALYs | 0.1687 (0.1393 to 0.1981) | 0.0150 | <0.001 |
|  |  | Deaths | 0.165 (0.1351 to 0.1949) | 0.0153 | <0.001 |
|  | 2021 | DALYs | 0.168 (0.1338 to 0.2022) | 0.0175 | <0.001 |
|  |  | Deaths | 0.1753 (0.1394 to 0.2113) | 0.0183 | <0.001 |
| Low SDI | 1990 | DALYs | 0.0901 (-0.0043 to 0.1844) | 0.0481 | 0.0706 |
|  |  | Deaths | 0.085 (-0.0103 to 0.1803) | 0.0486 | 0.0899 |
|  | 2021 | DALYs | 0.1695 (0.0848 to 0.2543) | 0.0432 | 0.0004 |
|  |  | Deaths | 0.173 (0.0834 to 0.2625) | 0.0457 | 0.0006 |
| Low-middle SDI | 1990 | DALYs | -0.0339 (-0.1001 to 0.0323) | 0.0338 | 0.3209 |
|  |  | Deaths | -0.0424 (-0.1109 to 0.0261) | 0.0349 | 0.2321 |
|  | 2021 | DALYs | -0.0109 (-0.0771 to 0.0554) | 0.0338 | 0.7493 |
|  |  | Deaths | -0.0146 (-0.0818 to 0.0527) | 0.0343 | 0.6735 |
| Middle SDI | 1990 | DALYs | 0.0724 (0.004 to 0.1409) | 0.0349 | 0.0447 |
|  |  | Deaths | 0.075 (0.0093 to 0.1407) | 0.0335 | 0.0310 |
|  | 2021 | DALYs | -0.0676 (-0.1372 to 0.002) | 0.0355 | 0.0644 |
|  |  | Deaths | -0.0561 (-0.1241 to 0.0118) | 0.0347 | 0.1135 |
| High-middle SDI | 1990 | DALYs | -0.0159 (-0.0331 to 0.0014) | 0.0088 | 0.0786 |
|  |  | Deaths | -0.0427 (-0.0606 to -0.0248) | 0.0091 | <0.001 |
|  | 2021 | DALYs | -0.0599 (-0.0798 to -0.04) | 0.0101 | <0.001 |
|  |  | Deaths | -0.0806 (-0.1013 to -0.0599) | 0.0106 | <0.001 |
| High SDI | 1990 | DALYs | -0.0417 (-0.0798 to -0.0037) | 0.0194 | 0.0379 |
|  |  | Deaths | -0.0305 (-0.0659 to 0.0048) | 0.0180 | 0.0988 |
|  | 2021 | DALYs | -0.0247 (-0.07 to 0.0206) | 0.0231 | 0.2924 |
|  |  | Deaths | -0.0077 (-0.0478 to 0.0324) | 0.0205 | 0.7078 |
